# Soluble Aβ pathology predicts neurodegeneration and cognitive decline independently on p‐tau in the earliest Alzheimer's *continuum*: Evidence across two independent cohorts

**DOI:** 10.1002/alz.14415

**Published:** 2025-02-03

**Authors:** Raffaele Cacciaglia, Carles Falcón, Gonzalo Sánchez Benavides, Anna Brugulat‐Serrat, Marta Milà Alomà, Marc Suárez Calvet, José Luis Molinuevo, Karine Fauria, Carolina Minguillón, Gwendlyn Kollmorgen, Clara Quijano‐Rubio, Kaj Blennow, Henrik Zetterberg, Luigi Lorenzini, Alle Meije Wink, Silvia Ingala, Frederik Barkhof, Craig W. Ritchie, Juan Domingo Gispert, for the ALFA study

**Affiliations:** ^1^ Barcelonaβeta Brain Research Center (BBRC), Pasqual Maragall Foundation Barcelona Spain; ^2^ Hospital del Mar Research Institute Barcelona Spain; ^3^ Centro de Investigación Biomédica en Red de Fragilidad y Envejecimiento Saludable (CIBERFES) Madrid Spain; ^4^ Centro de Investigación Biomédica en Red de Bioingeniería Biomateriales y Nanomedicina (CIBERBBN) Madrid Spain; ^5^ Global Brain Health Institute San Francisco California USA; ^6^ Northern California Institute for Research and Education San Francisco California USA; ^7^ Servei de Neurologia Hospital del Mar Barcelona Spain; ^8^ Roche Diagnostics GmbH Penzberg Germany; ^9^ Roche Diagnostics International Ltd. Rotkreuz Switzerland; ^10^ Department of Psychiatry and Neurochemistry Institute of Neuroscience and Physiology The Sahlgrenska Academy at the University of Gothenburg Mölndal Sweden; ^11^ Clinical Neurochemistry Laboratory Sahlgrenska University Hospital Mölndal Sweden; ^12^ UK Dementia Research Institute at UCL London UK; ^13^ Department of Neurodegenerative Disease UCL Institute of Neurology London UK; ^14^ Hong Kong Center for Neurodegenerative Diseases Hong Kong China; ^15^ Wisconsin Alzheimer's Disease Research Center University of Wisconsin School of Medicine and Public Health University of Wisconsin‐Madison Madison Wisconsin USA; ^16^ Department of Radiology & Nuclear Medicine Amsterdam UMC, Vrije Universiteit Amsterdam the Netherlands; ^17^ Queen Square Institute of Neurology and Centre for Medical Image Computing University College London London UK; ^18^ Edinburgh Dementia Prevention, Centre for Clinical Brain Sciences University of Edinburgh Edinburgh Scotland UK; ^19^ Universitat Pompeu Fabra Barcelona Spain; ^20^ Present address: Ottiliavej 9, 2500 København Denmark

**Keywords:** Amyloid beta, cerebrospinal fluid, medial temporal lobe, memory, p‐tau

## Abstract

**INTRODUCTION:**

Identifying the link between early Alzheimer's disease (AD) pathological changes and neurodegeneration in asymptomatic individuals may lead to the discovery of preventive strategies. We assessed longitudinal brain atrophy and cognitive decline as a function of cerebrospinal fluid (CSF) AD biomarkers in two independent cohorts of cognitively unimpaired (CU) individuals.

**METHODS:**

We used longitudinal voxel‐based morphometry (VBM) in combination with hippocampal subfield segmentation. Changes in neuroimaging and cognitive variables were inspected using general linear models (GLMs) adjusting by age, sex, apolipoprotein E (*APOE)* status, follow‐up time, and years of education.

**RESULTS:**

In both cohorts, baseline CSF amyloid beta (Aβ) biomarkers significantly predicted medial temporal lobe (MTL) atrophy rates and episodic memory (EM) decline independently of CSF phosphorylated tau (p‐tau).

**DISCUSSION:**

Our data suggest that soluble Aβ dyshomeostasis triggers MTL longitudinal atrophy and EM decline independently of CSF p‐tau. Our data underscore the need for secondary preventive strategies at the earliest stages of the AD pathological cascade.

**Highlights:**

- We assessed brain atrophy and cognitive decline in asymptomatic individuals.
- Aβ biomarkers predicted MTL atrophy independently of p‐tau.
- Our results underscore the importance of undertaking Alzheimer's preclinical trials.

## 1 BACKGROUND

Alzheimer's disease (AD), the most common form of dementia, is characterized by an insidious onset, where histopathological changes precede any detectable clinical symptoms by decades.^1^ Amyloid beta (Aβ) pathology is considered the earliest event in the pathophysiological cascade, which is later followed by neurofibrillary tau aggregation and neurodegeneration.^2^ About 20% of cognitively unimpaired (CU) individuals between 60 and 70 years of age are estimated to harbor Aβ pathology,^3^ and prevalence estimates are even higher with the occurrence of risk factors, such as older age, female sex, and carrying the apolipoprotein E (*APOE)* ε4 allele.^4^ Characterizing the link between early biomarker alterations and subsequent neurodegeneration in asymptomatic individuals is of critical importance, as it may lead to the discovery of effective preventive strategies before irreversible damage occurs. The medial temporal lobe (MTL) is the earliest target of the neurodegenerative process in AD.^5^ In this respect, previous cross‐sectional studies reported hippocampal atrophy in association with brain amyloidosis in CU individuals,^6^, ^7^, ^8^ while others have extended those findings to neocortical areas.^9^, ^10^, ^11^ However, other studies failed to establish a correlation^12^ and even observed increased cortical thickness (CTh).^13^ Discrepancies may be due to methodological factors or the difference in mean participants’ age across studies, as well as the biomarkers used for the definition of AD. Longitudinal studies provide greater accuracy in revealing these changes over time. In this regard, research conducted in CU individuals found that abnormal baseline Aβ levels significantly predicted gray matter volume (GMV) loss in MTL regions both when defining preclinical AD using cerebrospinal fluid (CSF)^14^ and positron emission tomography (PET)^15^, ^16^ biomarkers. Using a whole‐brain approach, others have reported a higher degree of cortical atrophy in posterior medial and medial temporal cortices in CU individuals with positive markers of Aβ.^17^, ^18^, ^19^ Yet, when accounting for the effects of both Aβ and tau, studies have reported that the detrimental effect of Aβ is significant only in the presence of abnormal tau.^20^, ^21^, ^22^ In addition, there is evidence that in CU individuals, the association between the burden of pathophysiology and the trajectories of the neurodegenerative process may be non‐linear and characterized by an initial volumetric or cortical thickness increase.^22^ While bringing a significant contribution to the field, all of the aforementioned studies are characterized by a relatively small sample size and a focus on elderly populations, when age‐related brain atrophy may already be evident. In addition, in later stages of the preclinical AD *continuum*, Aβ may have reached plateau levels^2^ and some degree of cortical atrophy due to early Alzheimer's pathological changes may have already occurred. In this study, we determined the impact of biomarkers of both Aβ and tau pathology quantified in the CSF on longitudinal changes of GMV in two independent large cohorts of CU individuals derived from the ALzheimer's and FAmilies (ALFA) and the European Prevention of Alzheimer's Disease (EPAD) studies. Alzheimer's pathophysiology was defined using soluble biomarkers to capture the earliest changes along the preclinical *continuum*, as CSF alterations precede those detected by PET.^23^ We used longitudinal voxel‐based morphometry (VBM) in combination with hippocampal subfield segmentation, taking advantage of ultra‐high‐resolution magnetic resonance imaging (MRI) sequences. In addition, changes in cognitive performance as a function of AD biomarkers were assessed.

## 2 METHODS

### 2.1 Study participants

The discovery sample included the first consecutive 353 participants recruited from the ALFA study^24^ (Clinicaltrials.gov Identifier: NCT01835717). For each study participant, baseline CSF and MRI acquisitions were performed less than 6 months apart. Twenty‐three participants exhibited abnormal CSF phosphorylated tau (p‐tau) with no evidence of Aβ pathology and were therefore excluded from further analyses, resulting in a final sample of 330 individuals. The confirmation sample included 371 participants from the EPAD study cohort.^25^ Twelve individuals with positive p‐tau and negative Aβ biomarker status were excluded, resulting in a final sample of 359 individuals. All participants had a Clinical Dementia Rating (CDR) scale = 0. Details on the study cohorts and the procedures for the *APOE* genotype, assessment of white matter hyperintensities (WMHs), and the Cardiovascular Risk Factors, Aging, and Incidence of Dementia (CAIDE) score are described in the online Supplementary Materials.

### 2.2 CSF sampling and analysis

For ALFA participants, CSF Aβ40, Aβ42, and neurofilament light chain (NfL) concentrations were determined using the NeuroToolKit, a panel of robust prototype biomarker assays, on cobas e601 (Aβ42) and e411 (Aβ40) instruments, while p‐tau181 was measured with the Elecsys Phospho‐Tau (181P) CSF immunoassay (Roche Diagnostics International Ltd., Rotkreuz, Switzerland) at the Clinical Neurochemistry Laboratory, University of Gothenburg, Sweden. To increase sensitivity, the ratio between Aβ42 and Aβ40 was calculated.^26^ ALFA individuals were considered Aβ positives (A+) if they had a value of CSF Aβ42/40 < 0.071 pg/mL and tau positives (T+) if they had a CSF p‐tau181 concentration >24 pg/mL.^27^ Details on CSF sampling in the ALFA cohort are provided in the Supplementary Materials. CSF sampling in EPAD participants was conducted using the fully automated Roche Elecsys instrument at the University of Gothenburg. Concentrations of Aβ42 and p‐tau181 were determined according to the manufacturer's instructions. EPAD individuals were categorized as Aβ positive (A+ group) if they had CSF Aβ42 concentrations <1000 pg/mL and tau positives (T+ group) if they had CSF p‐tau181 concentrations >27 pg/mL.^28^

### 2.3 Imaging data preprocessing

Procedures for imaging data acquisition are provided in the Supplementary Materials.

GMV changes were computed voxel‐wise using the pairwise longitudinal registration implemented in the statistical parametric mapping software (SPM, version 12) (https://www.fil.ion.ucl.ac.uk/spm/software/spm12/). The procedure involves a high‐dimensional warping between consecutive T1‐weighted scans, combining diffeomorphic and rigid‐body registrations and incorporating a correction for field inhomogeneities. For each subject, this procedure yields a parametric whole brain map encoding at each voxel the local tissue deformations that have occurred over time.^29^ Individual deformation maps were inspected for sample homogeneity using the computational anatomy toolbox (CAT12) (http://dbm.neuro.unijena.de/cat/) and normalized to the Montreal Neurological Institute (MNI) space using diffeomorphic image registration with DARTEL^30^ and spatially smoothed with an 8‐mm full width at half maximum (FWHM) Gaussian kernel before being submitted to group statistical analyses.

**RESEARCH IN CONTEXT**

1. **Systematic review**: We used PubMed to search for studies on the association between biomarkers of AD pathology, quantified both in the CSF and through molecular imaging and neurodegeneration. Results are heterogeneous and indicate that in asymptomatic individuals, amyloid impacts on gray matter atrophy only in the presence of abnormal tau.
2. **Interpretation**: Our data suggest that soluble Aβ pathology, the earliest AD pathophysiological alteration, is associated with neurodegeneration and cognitive decline, without the need for tau pathophysiology.
3. **Future directions**: This evidence underscores the relevance of anti‐amyloid therapies in CU individuals at the earliest stages of the Alzheimer's pathological cascade. Future studies will include more comprehensive measures of cognitive performance and perform multiple follow‐ups.

### 2.4 Automatic Segmentation of Hippocampal Subfields

Automatic Segmentation of Hippocampal Subfields (ASHS) software^31^ was used on the T1 and inversion recovery (IR) images to segment the hippocampal formation in the following sub‐regions: Brodmann areas 35 and 36, cornu Ammonis (CA) 1, 2 and 3, dentate gyrus (DG), entorhinal cortex (ERC), parahippocampus (PHC), subiculum (SUB), and sulcus (SUL). All segmentations were visually inspected before proceeding with the statistical analyses. The difference between baseline and follow‐up was computed for each subfield, to provide a measure of change over time. With this procedure, lower “delta” values indicate volumetric reduction over time.

### 2.5 Surface‐based analyses

In addition to longitudinal VBM, we also conducted vertex‐wise analyses using surface‐based morphometry (SBM) implemented in the Computational Analysis Toolbox (CAT12)^32^ to measure changes in cortical morphology over time as a function of AD biomarkers. SBM was employed to capture more subtle and localized cortical changes, particularly in cortical thickness, which may not be fully detectable with voxel‐wise methods, given its ability to account for the complex geometry of the cortical surface. First, baseline and follow‐up T1 scans were co‐registered and submitted to the longitudinal segmentation workflow provided in CAT12, with surface data being selected as additional output. Subsequently, individual surface data encoding CTh values at each vertex datum were resampled to a common surface template with 32,000 vertices and smoothed with a 12‐mm FWHM Gaussian kernel. Finally, we used the “cat_stat_diff” function to calculate the differential images between follow‐up and baseline for each participant. Finally, data were inspected with the sample homogeneity tool as provided in CAT12 and no outliers were detected.

### 2.6 Neuropsychological assessment

In ALFA, episodic memory (EM) was assessed using the Spanish validated version of the Free and Cued Selective Reminding Test (FCSRT),^33^ which produces the following variables: total recall (TR), total free recall (TFR), total delayed recall (TDR), and total delayed free recall (TDFR). In addition, we used the Preclinical Alzheimer Cognitive Composite (PACC), a cognitive assessment tool that was developed to detect subtle cognitive changes in individuals who may be in the preclinical stages of AD.^34^ Cognitive performance in EPAD was assessed through the Repeatable Battery for the Assessment of Neuropsychological Status (RBANS) test.^35^ The procedures for the FCSRT and RBANS are described in the Supplementary Materials. Similarly to the procedure applied to the hippocampal subfields, the difference between baseline and follow‐up was computed to provide a measure of change over time. With this procedure, lower “delta” values indicate volumetric reduction over time.

### 2.7 Statistical analyses

CSF AD biomarkers were treated both as categorical and continuous predictor variables in separate analyses. To assess AT biomarker group differences on GMV rates of change at the voxel level, we set up a general linear model (GLM) in SPM, where the normalized and smoothed divergence maps encoding the change over time were entered as dependent variables and AT status as predictor, with three levels (A–T–, A+T–, and A+T+). The following covariates were entered in the model: baseline age, sex, years of education, *APOE* ε4 status (ie, 0 = non‐carriers; 1 = carriers), follow‐up time, and total intracranial volume (TIV). In EPAD, dummy regressors coding the MRI scanner were additionally included. AT group differences were tested using pairwise *t*‐tests between each biomarker subgroup. In separate models, we entered the effects of continuous baseline CSF biomarkers, along with the covariates specified above. In both the categorical and continuous analyses, results were considered significant if surviving a voxel‐wise threshold of *p* < 0.005 with a cluster extent correction of *n* = 50 voxels. The same procedures were applied to the analyses of SBM data, with the only difference that TIV was not modeled as a covariate and cluster size was set to *n* = 15 vertices. The choice of adopting a slightly lower threshold was due to the inherent differences in spatial resolution between the two techniques. While VBM analyzes volumetric data and detects structural changes in larger clusters of voxels, SBM focuses on surface vertices, where changes are more localized. Therefore, a smaller threshold of 15 vertices was chosen for SBM to allow for the detection of subtle, spatially confined cortical changes, whereas a threshold of 50 voxels was applied in VBM to account for the broader, more diffuse nature of voxel‐wise analysis. Next, we determined the impact of CSF biomarkers on longitudinal hippocampal subfield volume (HSV) and cognitive performance using linear mixed‐effects (LME) modeling in R version 4.3.0 (R Foundation for Statistical Computing, Vienna, Austria. https://www.R‐project.org/), with subject‐specific intercepts, adjusted for baseline age, sex, years of education, and *APOE* ε4 genotype (ie, binary status). For HSV analyses, TIV was further added as a covariate. LME analyses were performed using both categorical (AT status, three levels) and continuous biomarker predictors. In this latter case, CSF Aβ42/40, p‐tau181, and NfL were included in the same models. In the ALFA sample, we performed additional models adjusted for CAIDE risk scores and WMH. To obtain further confirmation of the impact of CSF markers on both hippocampal structures and changes in cognition, we also performed complementary analyses using linear regression, selecting the difference score between consecutive visits as dependent variables (please refer to the Supplementary Materials for details).

## 3 RESULTS

### 3.1 Samples’ characteristics

Demographic characteristics at baseline of the two samples are presented in Table 1. Compared to ALFA, EPAD participants were significantly older, harbored a lower proportion of *APOE* ε4 allele and a lower proportion of CSF Aβ42 positive individuals. Within the ALFA sample, mean age and years of education significantly differed among AT status subgroups. Bonferroni corrected post‐hoc comparisons revealed that the A+T+ were older than the A–T– individuals (*p* = 0.001), and that, compared to both A–T– and A+T–, A+T+ individuals were less educated (*p* = 0.023) (Table S1). Similarly, in EPAD, AT status had a significant effect on age, where the A+T+ was significantly older compared with both the A–T– (*p* = 0.001) and the A+T– groups (*p* = 0.001) (Table S2).

**TABLE 1:** Baseline participant characteristics.

	ALFA (*n* = 330)	EPAD (*n* = 359)	*p*‐value
Age, y	60.85 (4.74)	66.17 (6.59)	<0.01
Sex, f	195 (59.1%)	192 (53.5%	0.13
Education, y	13.54 (5.51)	14.17 (3.73)	0.02
*APOE* ε4 carrier, *n*	177 (53.6%)	123 (34.3%)	<0.01
CSF Aβ42, pg/mL	1270.54 (531.35)	1307.17 (549.51)	0.63
CSF p‐tau181, pg/mL	15.82 (6.73)	17.55 (7.63)	0.72
CSF Aβ42 positive,^a^ *n*	143 (43.3%)	125 (34.8%)	0.02
CSF Aβ42/40 positive, *n*	115 (34.8%)		
Follow‐up time, y	3.35 (0.53)	1.01 (0.08)	<0.01
MMSE	29.18 (0.93)	28.74 (1.62)	<0.01
MMSE range	27‐30	19‐30	
TIV, mm^3^	1431.28 (153.12)	1490.23 (148.51)	<0.01

*Note*: Data are presented as mean (SD) or *n* (%)

Abbreviations: Aβ, amyloid beta; *APOE*, apolipoprotein‐E; CSF, cerebrospinal fluid; f, female; MMSE, Mini‐Mental State Examination; p‐tau181, phosphorylated tau; TIV, total intracranial volume; y, years.

^a^ The cut‐off value of CSF Aβ42 in ALFA was determined at 1098 pg/mL, and data are shown for comparison with EPAD study cohort exclusively.

### 3.2 GMV changes atrophy across the preclinical AT stages using longitudinal VBM

**FIGURE 1 alz14415-fig-0001:**
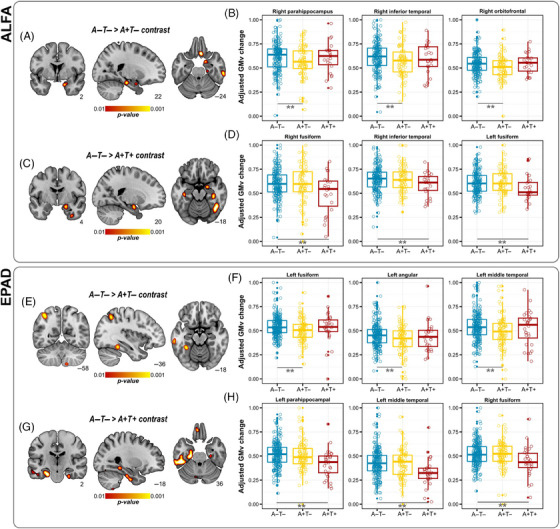
Longitudinal GMV changes across different stages of preclinical Alzheimer's. (A) Volume rendering of brain regions surviving statistical threshold, showing a higher degree of cortical atrophy in the A+T− compared to A−T− subgroup in the ALFA sample. (B) Group boxplot in exemplary brain regions representing statistical contrast as in panel A. (C) Volume rendering of brain regions surviving statistical threshold, showing a higher degree of cortical atrophy in A+T+ compared to A−T− subgroup in ALFA sample. (D) Group boxplot in exemplary brain regions representing statistical contrast as in panel C. (E) Volume rendering of brain regions surviving statistical threshold, showing a higher degree of cortical atrophy in A+T− compared to A−T− subgroup in EPAD sample. (F) Group boxplot in exemplary brain regions representing statistical contrast as in panel E. (G) Volume rendering of brain regions surviving statistical threshold, showing a higher degree of cortical atrophy in A+T+ compared to A−T− subgroup in EPAD sample. (H) Group boxplot in exemplary brain regions representing statistical contrast as in panel G. In all boxplots, plotted values represent mean computed across all voxels in significant clusters, and values are adjusted for age, sex, years of education, and *APOE* ε4 genotype. **p < 0.005, with *n* = 50 cluster correction at voxel level. AD, Alzheimer's disease; EPAD, European Prevention of Alzheimer's Disease; GMV, gray matter volume.

In ALFA, the A+T– group showed a faster degree of GMV atrophy in the right inferior temporal gyrus (ITG), right middle temporal gyrus (MTG), orbitofrontal gyrus (OFG), right parahippocampal gyrus (PHG), and the left posterior hippocampus (HC) compared to A–T– (Figure 1A,B, Table S3). Compared to A–T–, the A+T+ group displayed greater atrophy rates in the bilateral fusiform gyrus (FG), right ITG, right HC, and the right amygdala (Figure 1C,D, Table S3). Finally, compared to A+T–, the A+T+ individuals showed greater GMV atrophy in the right FG and right HC (Table S3). These results remained significant when adjusting by WMH and CAIDE score (Table S4). In EPAD, the A+T– group showed greater atrophy rates in the left FG, angular gyrus (AG), and left MTG compared to A–T– (Figure 1E,F, Table S5). Compared to A–T–, the A+T+ group showed GMV reductions in the left MTG, right FG, and bilateral PHG (Figure 1G,H, Table S5). Finally, compared to A+T–, the A+T+ individuals showed greater GMV atrophy in the left ITG (Table S5).

### 3.3 Impact of continuous CSF biomarkers on GMV changes

**FIGURE 2:**
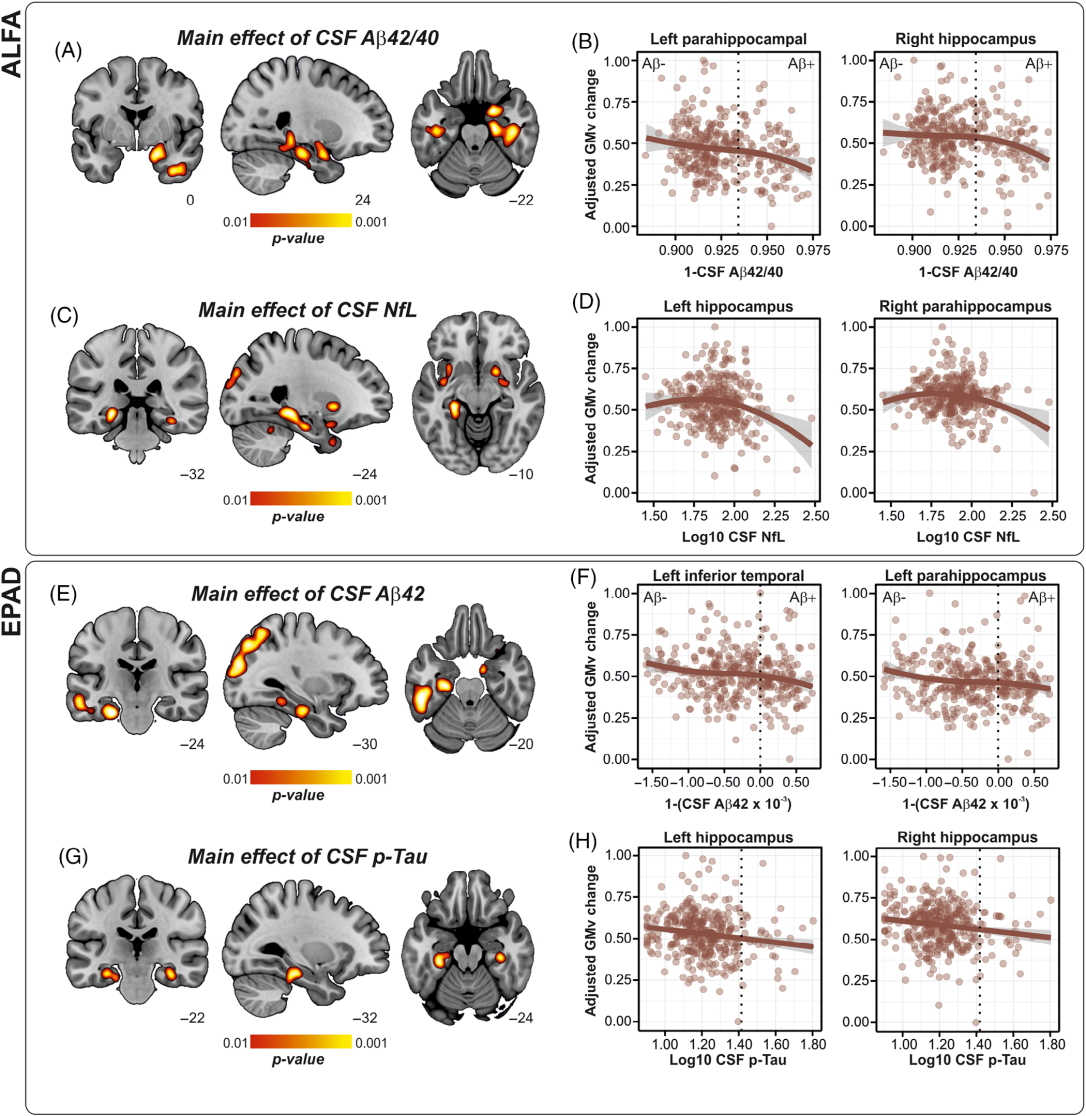
Effects of continuous baseline CSF biomarker concentration on longitudinal GMV changes. (A) Volume rendering of significant regions where a main effect of continuous CSF Aβ42/40 was predictive of longitudinal GMV atrophy in ALFA sample. (B) Scatterplot in exemplary regions as in panel A. Dashed line indicates threshold for CSF Aβ42/40 positivity. (C) Volume rendering of significant regions where a main effect of continuous CSF NfL was predictive of longitudinal GMV atrophy in ALFA sample. (D) Scatterplot in exemplary regions as in panel C. (E) Volume rendering of significant regions where a main effect of continuous CSF Aβ42 was predictive of longitudinal GMV atrophy in EPAD sample. (F) Scatterplot in exemplary regions as in panel E. Dashed line indicates threshold for CSF Aβ42 positivity. (G) Volume rendering of significant regions where a main effect of continuous CSF p‐tau181 was predictive of longitudinal GMV atrophy in EPAD sample. (H) Scatterplot in exemplary regions as in panel G. Dashed line indicates threshold for CSF p‐tau181 positivity. For illustrative purposes, regressions lines in scatterplots are fitted using a loess function. Shaded area indicates 95% confidence intervals. In all scatterplots, plotted values represent mean computed across all voxels in significant clusters, and values are adjusted by age, sex, years of education, and *APOE* ε4 genotype. Aβ, amyloid beta; CSF, cerebrospinal fluid; EPAD, European Prevention of Alzheimer's Disease; GMV, gray matter volume; NfL, neurofilament light chain; p‐tau, phospho‐Tau.

In ALFA, CSF Aβ42/40 predicted longitudinal GMV atrophy in the bilateral ITG, as well as the bilateral[Fig alz14415-fig-0001] HC (Figure 2A,B). No significant main effects were observed for CSF p‐tau181, while CSF NfL concentrations predicted atrophy in the bilateral anterior and posterior HC (Figure 2C,D). These results were still significant after adjusting for the effect of CAIDE score and WMH (Table S6). To rule out any potential influence of incipient tau pathology, analyses were further repeated within the A+T– subgroup (*n* = 88). In this subsample, baseline CSF Aβ42/40 still predicted longitudinal GMV atrophy in the right FG, left ITG, left anterior HC, and left PHG, while CSF NfL significantly predicted GMV atrophy in the bilateral PHG and left HC (Figure S1a,b). In EPAD, baseline CSF Aβ42 significantly predicted longitudinal GMV atrophy in the left HC, left PHG, left ITG, and the precuneus (PCN) (Figure 2E,F), while CSF p‐tau181 predicted GMV atrophy in the bilateral HC (Figure 2G,H). Restricting the analyses to the A+T– subgroup (*n* = 97) yielded a significant main effect of CSF Aβ42 in predicting GMV atrophy in the left PHG as well as the left AG, with these results being still significant after adjusting by CSF pTau (Figure S1c).

#### 3.3.1 SBM results in ALFA sample

Compared to A–T–, the A+T– individuals showed accelerated CTh thinning in the right MTG and ITG, while the A+T+ showed significantly faster atrophy rates in the MTG. Finally, compared to the A+T–, the A+T+ group showed accelerated atrophy rates in the left FG (Table S7 and Figure S2a,c). When looking at continuous biomarkers, baseline CSF Aβ42/40 predicted longitudinal CTh reduction in the right PHG, while CSF p‐tau was related to cortical thinning in the right MTG, ITG, and left FG. Finally, CSF NfL predicted CTh reduction in the left superior temporal gyrus (STG), right superior occipital gyrus (SOP), and right AG (Table S8 and Figure S3a,c). Within the A+T– subsample, CSF Aβ42/40 predicted longitudinal CTh reduction in the left MTG and PCN, as well as the right ITG and temporal pole (TP). CSF p‐tau was not significantly related to a decreased CTh in any region. Finally, CSF NfL concentrations significantly predicted CTh thinning in the left inferior occipital gyrus and the middle cingulate cortex (Table S9 and Figure S3d,f).

#### 3.3.2 SBM results in EPAD sample

No significant differences could be observed between A+T– and A–T–. Compared to A–T–, the A+T+ showed a steeper CTh in the right superior parietal gyrus (SPG), as well as the left PHG, ITG, inferior frontal gyrus (IFG), and PCN. Similarly, compared to the A+T–, the A+T+ displayed significantly steeper CTh atrophy in the right SPG, right FG, and the left ITG, MTG, and PHG (Table S10 and Figure S2d,f). When looking at continuous biomarkers, baseline CSF Aβ42 predicted longitudinal CTh reduction in the bilateral AG, as well as the right ITG, and the left PHG and PCN. CSF p‐tau predicted CTh thinning in the bilateral IFG, as well as the left ITG, right AG, and left FG (Table S11 and Figure S4a,b). Within the A+T– subsample, we found that CSF Aβ42 significantly predicted CTh thinning in the right middle frontal gyrus (MFG), IFG, PHG, and AG, as well as the left insula (INS) and PCN. Within this subsample, CSF p‐tau predicted CTh thinning in the right IFG, right STG, and right ITG (Table S12 and Figure S4c,d).

### 3.4 Impact of CSF biomarkers on hippocampal subfields in ALFA sample

**FIGURE 3:**
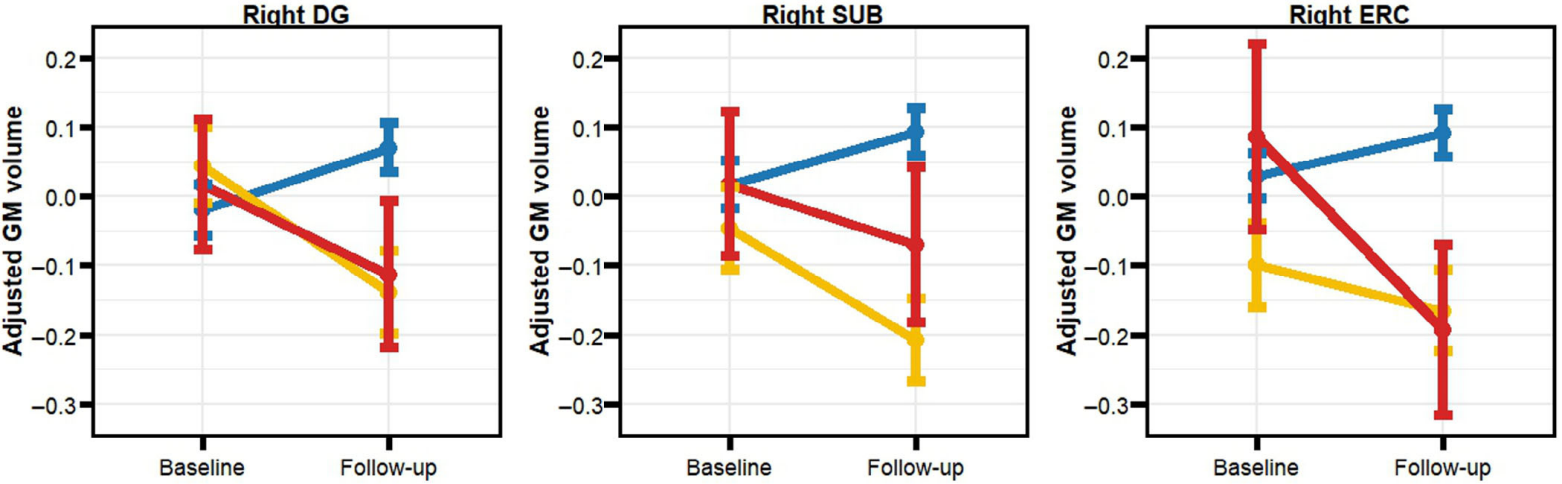
Hippocampal subfield longitudinal GMV changes as function of AT status. The longitudinal GMV trajectories of the right DG, right SUB, and ERC were significantly different as a function of AT status in ALFA sample. Error bars represent standard error of the mean. Values are adjusted for age, sex, years of education, and *APOE* ε4 genotype. DG, dentate gyrus; ERC, entorhinal cortex; GMV, gray matter volume; SUB, subiculum.

LME analyses revealed a significant interaction between follow‐up time and AT status for the right DG and SUB, indicating higher atrophy rates for the A+T– compared to the A–T–. In addition, the A+T+ compared to the A–T– showed a faster volume reduction in the right ERC (Figure 3 and Table 2). When modeling CSF biomarkers continuously, we found that baseline CSF Aβ42/40 concentrations significantly predicted longitudinal atrophy in the bilateral CA1, right DG, SUB, and BA35 (Table 2). For CSF pTau and NfL no significant effects were observed. When repeating the analyses in the A+T– individuals, we found significant interactions between baseline CSF Aβ42/40 and time, indicating an effect of the biomarker over the longitudinal atrophy of the right SUB and the left CA1. Additional analyses conducted using linear regression models over differential hippocampal subfield volumes (ie, follow‐up *minus* baseline), confirmed the foregoing results (Tables S13 and S14).

**TABLE 2:** Impact of baseline CSF biomarker status on hippocampal subfields longitudinal atrophy patterns in ALFA sample.

Model	Interaction term	Hippocampal subfield	Parameter Estimate	SEM	*t*‐value	*p*‐value
**Categorical biomarker**	**AT status × Time (A**–**T**– **vs A+T‐)**	Right DG	−5.04	1.93	−2.65	0.009
	**AT status × Time (A**–**T**– **vs A+T+)**	Right SUB	−2.71	0.98	−2.77	0.005
	**AT status × Time (A**–**T**– **vs A+T+)**	Right ERC	−5.82	2.57	−2.26	0.02
**Continuous biomarker**	**CSF Aβ42/40 × Time**	Right CA1	168.26	55.51	3.03	0.002
		Left CA1	134.83	54.95	2.45	0.01
		Right DG	114.12	47.51	2.41	0.01
		Right SUB	63.33	24.54	2.58	0.01
		Right BA35	97.49	35.31	2.76	0.006

Abbreviations: A, amyloid beta status; BA35, Brodmann area 35; CA1, cornu ammonis 1; DG, dentate gyrus; ERC, entorhinal cortex; SEM, standard error of the mean; SUB, subiculum; T, p‐tau status.

### 3.5 Impact of CSF markers on longitudinal cognition

**FIGURE 4:**
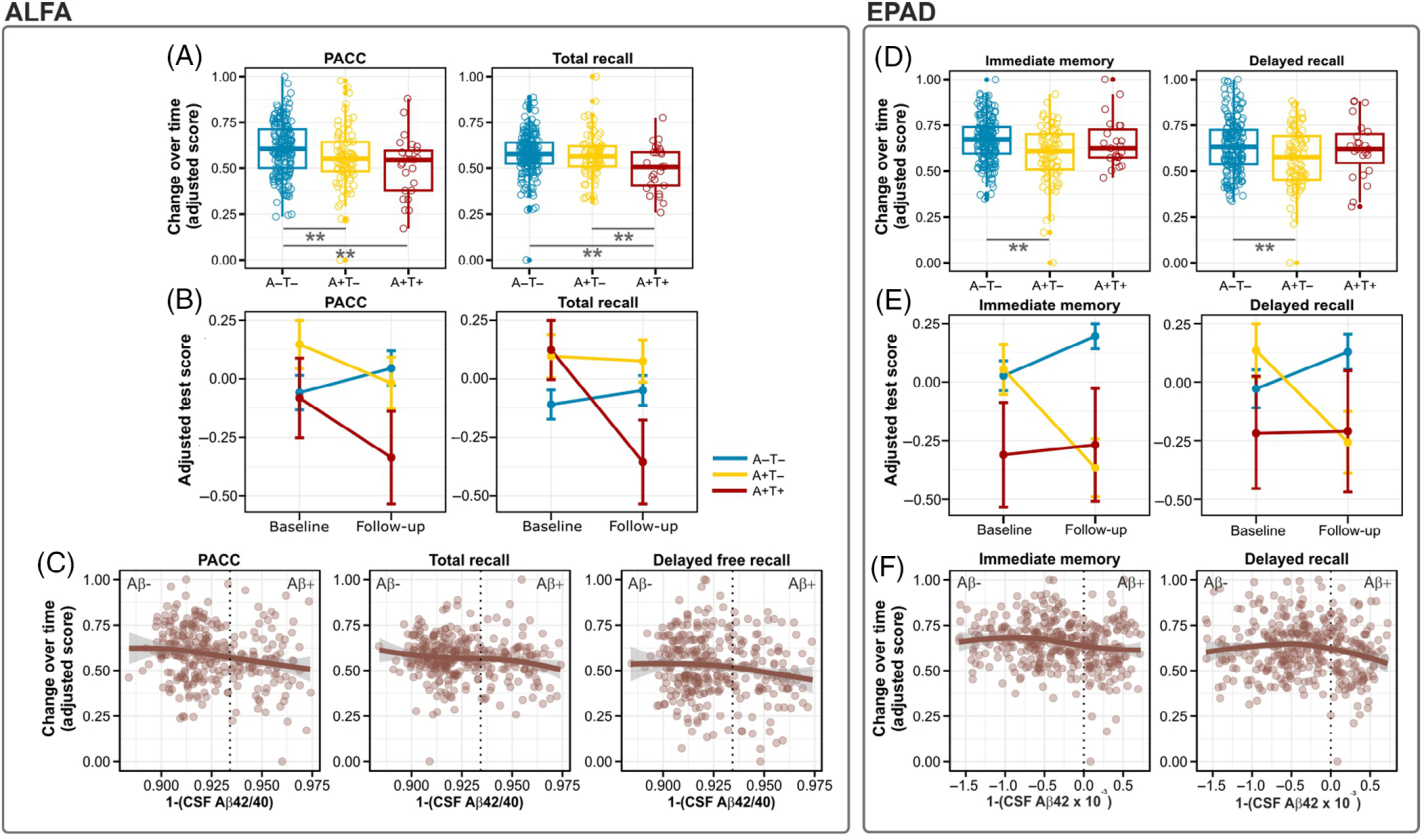
Impact of CSF AD biomarkers on longitudinal cognitive decline. (A) Group boxplots showing significant impact of AT status on longitudinal PACC and the TR of the FCSRT in the ALFA sample. (B) Line plots showing that the longitudinal trajectory in both PACC and TR was significantly affected by AT status. (C) Scatterplots showing significant cognitive decline in PACC, TR, and delayed free recall as a function of baseline CSF Aβ42/40 concentrations. (D) Group boxplots showing significant impact of AT status on longitudinal immediate memory and delayed recall in EPAD sample. (E) Line plots showing that the longitudinal trajectory in both immediate memory and delayed recall was significantly affected by AT status. (F) Scatterplots showing significant cognitive decline in both cognitive domains as a function of baseline CSF Aβ42 concentrations. Shaded area indicates 95% confidence intervals. In all boxplots and scatterplots, plotted values represent mean computed across all voxels in significant clusters, and values are adjusted for age, sex, years of education, and *APOE* ε4 genotype. AD, Alzheimer's disease; Aβ, amyloid beta; CSF, cerebrospinal fluid; EPAD, European Prevention of Alzheimer's Disease; FCSRT, Free and Cued Selective Reminding Test; PACC, Preclinical Alzheimer Cognitive Composite; TR, total recall.

In the ALFA sample, we found a significant interaction between AT status and time, indicating a steeper PACC decline in both the A+T– and A+T+ subgroups compared with the A–T– subgroup. In addition, a significant interaction with the TR index of the FCSRT indicated that the A+T– subgroup exhibited a faster decline compared to the A–T– subgroup (Figure 4A,B and Table S15). When modeling CSF biomarkers continuously, we found that, over time, CSF Aβ42/40 was significantly related to PACC decline as well as the total and delayed free recall indices of the FCSRT (Figure 4C and Table S16), while no significant effects were found for CSF p‐tau181 (Figure S5). In EPAD, the significant interaction between AT status and time indicated a steeper cognitive decline for both immediate and delayed recall in A+T– compared to A–T– (Figure 4D,E and Table S17). When modeling CSF biomarkers continuously, CSF Aβ42 significantly predicted longitudinal decline in immediate recall and delayed recall (Figure 4F and Table S18). As in the ALFA sample, no significant effects were found for CSF p‐tau181 (Figure S6). Results of the analyses conducted in the A+T– subgroup in each study sample yielded no significant results. Additional analyses conducted using linear regression models over differential cognitive scores in both the ALFA and EPAD samples (ie, follow‐up *minus* baseline) confirmed the above results (Supplementary Materials).

## 4 DISCUSSION

We reported that soluble Aβ pathology predicts longitudinal GMV atrophy in brain regions vulnerable to early Alzheimer's neurodegeneration, such as the MTL areas. These results were consistent in two independent cohorts and were independent of sex, age at baseline, follow‐up time, and CSF p‐tau181 levels. In the ALFA sample, the results also held after adjusting for markers of axonal injury, small vessel disease, and cardiovascular risk factors, which are known contributors to neurodegeneration.^36^, ^37^, ^38^ This additional evidence suggests that the observed MTL atrophy is primarily driven by Alzheimer's‐related pathology rather than non‐specific risk factors for neurodegeneration. Furthermore, these results were replicated in subsamples negative for CSF p‐tau181 markers (A+T– subgroup), indicating that Aβ pathology triggers MTL atrophy even when CSF p‐tau levels are within normal ranges.

It is important to highlight that we employed two complementary approaches to quantify atrophy rates at the whole‐brain level: longitudinal VBM and SBM. The rationale for using both techniques lies in their ability to capture different aspects of brain morphology. Longitudinal VBM measures volumetric changes, while SBM offers detailed insights into cortical surface structure and CTh. In this study, these two methods yielded consistent results, and their combined use provided valuable insights into the evolution of atrophy patterns during the early stages of preclinical AD. Paralleling our findings on structural brain atrophy, we demonstrated that baseline CSF Aβ significantly predicted longitudinal EM decline independently of CSF p‐tau181 in both cohorts. In the ALFA cohort, we additionally observed a significant decline in the PACC score as a function of CSF Aβ42/40, in models adjusted for p‐tau181 and NfL.

Our findings are in line with previous studies that found increased atrophy rates in the MTL areas in CU individuals with positive CSF Aβ markers, compared to Aβ‐negative individuals.^14^, ^17^ For example, previous research reported increased atrophy rates in MTL regions over a 12‐month follow‐up period. In addition, studies using PET imaging markers have shown significant associations between baseline Aβ burden and the rate of atrophy in both inferior and medial temporal regions,^15^ as well as more extended temporal areas.^18^ However, these studies did not provide a concomitant assessment of tau pathology, making it difficult to attribute the observed atrophy rates solely to Aβ pathology. In contrast, our study addressed this limitation by including tau pathology as a covariate in the models. Desikan et al.^20^ reported that among CU individuals, baseline CSF Aβ42 predicted EC atrophy over 48 months, but only in the presence of abnormal CSF p‐tau181 levels. Similarly, Xie et al.^21^ found significantly higher MTL atrophy rates only in individuals with both abnormal Aβ and tau, but not in those with evidence of Aβ pathology alone. Our data challenge these previous findings, suggesting that Aβ can drive MTL neurodegeneration even in the absence of abnormal tau. It is important to emphasize that both the ALFA and EPAD cohorts were younger than those in many previous studies, and the proportions of individuals with abnormal CSF p‐tau181 were relatively low (8.18% in ALFA and 7.79% in EPAD). Thus, our study population represents individuals at very early stages of the Alzheimer's pathophysiological *continuum*, which may have enabled us to detect subtle changes in MRI and cognition that occur earlier in the preclinical biomarker cascade.

In addition to our findings related to Aβ pathology, we also reported CSF p‐tau‐related GMV atrophy in both cohorts, consistent with previous literature. Furthermore, the aforementioned studies had relatively small sample sizes compared to our study, which included larger cohorts. Another potential source of discrepancy between our results and those of previous studies may be the methodology employed. We implemented a longitudinal voxel‐wise registration of serial MRI scans using a high‐dimensional warping procedure and performed hippocampal subfield segmentation using ultra‐high‐resolution MRI sequences. These advanced imaging techniques may provide greater sensitivity when detecting small effects at the group level compared to conventional region‐of‐interest (ROI) analyses.

When examining hippocampal subfields, we found that baseline CSF Aβ42/40 predicted longitudinal atrophy in the bilateral CA1, right DG, SUB, and Brodmann area 35 (BA35). Previous studies showed that CA1 and DG volumes correlate with clinical prognosis in AD patients^39^, ^40^ and hippocampal memory formation,^41^ respectively. Furthermore, when modeling the slopes of GMV changes in hippocampal subfields, we observed an initial increase in volume only in individuals with no evidence of Alzheimer's pathology (A–T–). By contrast, individuals in both the A+T– and A+T+ groups exhibited significant atrophy over time. This observation is consistent with models proposing a non‐linear relationship between AD pathology and brain morphology in preclinical stages of the disease.^22^, ^42^ Other studies have similarly reported increased cerebral glucose metabolism in CU individuals as a function of Aβ pathology in temporal areas.^43^ While several mechanisms may explain this non‐linear pattern, it has been suggested that an initial neuroinflammatory response to Aβ fibrils may induce transient GMV hypertrophy in MTL regions.^44^ Evidence suggests that both microglia and astroglia react to early Aβ deposition^45^, ^46^ and that the HC is a brain region particularly susceptible to neuroinflammation,^47^, ^48^ with prominent activity‐dependent synaptic plasticity.^49^

Our finding of an Aβ‐related impact on MTL atrophy in the absence of tau pathology deserves further discussion. Aβ and tau proteinopathies have distinct topological distributions, with Aβ first depositing in medial frontal and posteromedial cortical areas,^50^, ^51^ while tau begins to aggregate in the MTL.^52^ Several mechanisms could underlie our results. First, CSF markers reflect the soluble Aβ pool, capturing the imbalance between Aβ42 production and clearance.^53^ Unlike PET imaging, CSF Aβ markers may better reflect the presence of cerebral Aβ oligomers,^54^, ^55^ which are more neurotoxic than fibrillary plaques.^56^ Aβ oligomers can also induce neuronal apoptosis in regions distal to their production site, particularly the HC.^57^, ^58^, ^59^ Another possible mechanism involves the *APOE* ε4‐risk allele. We recently showed that *APOE* ε4 modulated the relationship between CSF and PET Aβ markers, promoting greater Aβ accumulation in MTL regions for a given level of soluble Aβ.^60^ This suggests that *APOE* ε4 carriers may have greater hippocampal Aβ deposition, leading to hyperexcitability and degeneration over time.^61^ While the potential interaction between Aβ burden and *APOE* ε4 in determining hippocampal atrophy trajectories was beyond the scope of this study, it warrants further investigation in future research.

Although, compared to previous studies,^14^, ^17^, ^20^, ^21^, ^22^ our overall sample size was larger, one limitation of our study concerns the relatively low proportion of individuals with evidence of tau pathology (8.18% in ALFA and 7.79% in EPAD). This factor limits the generalizability of our findings. Future studies investigating longitudinal atrophy patterns in preclinical AD should employ more balanced sample sizes across different stages of pathology. Another limitation is the use of CSF p‐tau181 as a surrogate marker for tau pathology. Although p‐tau181 has been widely used as AD biomarker,^1^, ^62^ recent studies identified other epitopes, such as p‐tau217^63^ and p‐tau231,^64^ which have demonstrated greater sensitivity in detecting tau pathology. Incorporating these epitopes will be crucial in future longitudinal studies of preclinical AD.

Moreover, it is important to consider that the A/T/N staging system has limitations due to the low concordance between the selected biomarkers, especially when they are fluid (eg, CSF) or neuroimaging markers (eg, MRI or PET).^65^, ^66^ This could lead to a misclassification of our subjects. However, it is important to highlight that we classified participants based solely on A and T, not N, which, among the three, appears to be the category with the highest degree of inconsistency among biomarkers.^65^, ^66^

Another challenge is the variability in follow‐up periods between cohorts, which may introduce inconsistencies in the comparison of cortical atrophy progression rates. Future studies with more harmonized follow‐up durations will be important for validating the predictive role of Aβ across different time windows. Additionally, we relied on a limited subset of core CSF biomarkers to define the degree of underlying Alzheimer's pathology. However, the complexity of AD involves additional processes, such as synaptic dysfunction, neuroinflammation, and other neurodegenerative mechanisms.^27^ Future research should incorporate a broader panel of biomarkers, including synaptic and glial markers, to better account for the multifaceted nature of disease progression. Moreover, we acknowledge the importance of considering Aβ42 not only as a marker of Aβ deposition but also as a potential indicator of broader neurodegenerative processes, including synaptic loss and neuroinflammation, as suggested in previous studies.^67^

Finally, the characteristics of our sample, composed of middle‐aged CU individuals, even though they were selected as a population at high risk to develop AD,^24^, ^25^ may limit the generalizability of our findings to older populations, where AD is more prevalent.

In conclusion, we demonstrated in CU individuals that baseline CSF Aβ biomarkers significantly predicted GMV atrophy in MTL and other cortical regions, along with EM decline. These findings were independent of soluble tau levels, suggesting that incipient cerebral Aβ is neither part of normal aging nor a benign process. Our data foster the need for preventive interventions in asymptomatic individuals with evidence of soluble Aβ pathology in secondary prevention trials. At‐risk CU individuals may benefit from multimodal strategies that shall combine both pharmacological and non‐pharmacological interventions, as both have been shown to be successful in reducing Aβ pathology.^68^, ^69^, ^70^, ^71^

## CONFLICT OF INTEREST STATEMENT

This publication is part of the ALFA study (ALzheimer's and FAmilies). The authors would like to express their most sincere gratitude to the ALFA project participants, without whom this research would not have been possible. The authors would like to thank Roche Diagnostics International Ltd. for kindly providing the kits for the CSF analysis of ALFA+ participants. F.B. is Steering Committee or Data Safety Monitoring Board member for Biogen, Merck, ATRI/ACTC, and Prothena, has served as consultant for Roche, Celltrion, Rewind Therapeutics, Merck, IXICO, Jansen, and Combinostics, has research agreements with Merck, Biogen, GE Healthcare, Roche, and is co‐founder and shareholder of Queen Square Analytics LTD. J.D.G. has received research funding by GE Healthcare, Roche Diagnostics and Hoffmann – La Roche, speaker's fees from Biogen and Philips Nederlands, and consulting fees from Roche Diagnostics and serves on the Molecular Neuroimaging scientific board of Prothena Biosciences. The rest of the authors have no conflict of interest to declare. Author disclosures are available in the supporting information.

## CONSENT STATEMENT

All participants provided written informed consent.

## Supporting information



Supporting information

Supporting information
