# Supplementary material for: Soluble Aβ pathology predicts neurodegeneration and cognitive decline independently on p‐tau in the earliest Alzheimer's continuum: Evidence across two independent cohorts
Source: Alzheimers Dement. 2025 Feb 3;21(2):e14415. doi: 10.1002/alz.14415 (PMC11848178; doi:10.1002/alz.14415)
Supplement: Supplementary file 1 — Supporting information [file ALZ-21-e14415-s002.docx]

**Supplementary Materials**

**Title:** Soluble Aβ pathology predicts neurodegeneration and cognitive decline independently on p-tau in the earliest Alzheimer’s *continuum*: evidence across two independent cohorts

**Authors:** Raffaele Cacciaglia, Carles Falcón, Gonzalo Sánchez Benavides, Anna Brugulat-Serrat, Marta Milà Alomà, Marc Suárez Calvet, José Luis Molinuevo, Karine Fauria, Carolina Minguillón, Gwendlyn Kollmorgen, Clara Quijano-Rubio, Kaj Blennow, Henrik Zetterberg, Luigi Lorenzini, Alle Meije Wink, Silvia Ingala, Frederik Barkhof, Craig W. Ritchie and Juan Domingo Gispert, for the ALFA study

**Supplementary Methods**

***Study Participants***

All subjects signed the study's informed consent which was approved by the Independent Ethics Committee “Parc de Salut Mar,” Barcelona and was conducted according to the Declaration of Helsinki.

***APOE genotype***

For ALFA participants, total DNA was obtained from blood cellular fraction by proteinase K digestion followed by alcohol precipitation. For EPAD participants, genomic DNA was isolated from whole blood and genotyping was performed in 384 well-plates, using the TaqMan polymerase chain reaction-based method. Both samples were genotyped for two single nucleotide polymorphisms (SNPs), rs429358 and rs7412 to define the *APOE*-ε2, ε3 and ε4 alleles. For both cohorts, subjects were classified as ε4 carriers (one or two alleles) or non-carriers.

***Imaging data acquisition***

In ALFA, a high-resolution T1-weighted 3D-TFE sequence was acquired at baseline and follow-up with a 3T Philips Ingenia CX scanner with the following sequence parameters: voxel size = 0.75 mm isotropic, field of view (FOV) = 240 x 240 x 180 mm^3^, flip angle = 8º, repetition time = 9.9 ms, echo time = 4.6 ms, TI = 900 ms. In addition, a ultra-high resolution dual-echo inversion recovery (IR) 3D sequence centered in the hippocampus was acquired with the following parameters: voxel size = 0.39 x 0.39 x 2.00 mm, TR = 2.68s, TE = 0.26 s; TI = 0.4 s; flip angle = 90ºm FOV = 576x576. The EPAD imaging protocol includes MRI acquisition from different MRI centers and it has been described elsewhere (Lorenzini et al., 2022). The current study included images collected in 16 different centers.

***Assessment of cardiovascular risk factors and white matter hyper-intensities***

In the ALFA study cohort, statistical models were further adjusted by cardiovascular risk factors and white matter hyper-intensities (WMH). The Cardiovascular Risk Factors, Aging, and Incidence of Dementia (CAIDE) risk score (Kivipelto et al,. 2006), which considers age, education, sex, systolic blood pressure, body mass index (BMI), total cholesterol, and physical activity as risk factors. WMH segmentation was performed using Bayesian Model Selection (BaMoS), a hierarchical fully-unsupervised model selection framework (Sudre et al., 2015). Regional values of WMH were obtained by averaging lesions within regions taking into account lobar boundaries (frontal, parietal, temporal, and occipital) and distance between the ventricular surface and cortex (4 layers). WMH regional values were averaged between the left and right hemisphere.

***CSF sampling procedures in the ALFA cohort***

CSF was collected into a 15mL sterile polypropylene sterile tube (Sarstedt. Nümbrecht. Germany; cat. no. 62.554.502). CSF was aliquoted in volumes of 0.5mL into sterile polypropylene tubes (0.5mL Screw Cap Micro Tube Conical Bottom; Sarstedt. Nümbrecht. Germany; cat. no. 72.730.005) and immediately frozen at −80°C. Overall, the time between collection and freezing was less than 30 minutes. All the determinations were done in aliquots that had never been previously thawed.

***Episodic memory assessment in the ALFA cohort***

In both cohorts, general cognitive ability was measured with the Mini-Mental State Examination (MMSE).

The Free and Cued Selective Reminding Test (FCSRT) consists of the learning and retention of a list of 16 semantically unrelated words through a controlled learning process that uses semantic encoding. During the initial learning phase, participants are asked to read aloud 16 printed words grouped in four cards (4 words in 4 cards) and associate them to their corresponding semantic cue (e.g., “Which is the bird?”). After this encoding procedure, three recall trials follow, each one preceded by a number subtraction task of 20s of duration. Each trial consists of a free recall followed by a cued recall of the words not spontaneously retrieved, by using the semantic cues provided previously. The words that are not recalled after cueing are selectively reminded in the two initial trials,

but not in the last one. A delayed free and cued recall is performed after 25–35 min. A complete description of the items used in the FCSRT is provided in Grau-Guinea et al., (2021). Here below, we provide a description of the 4 variables produced by the FCSRT: 1) Total Free Recall, indexing the sum of the words correctly retrieved in the three free recall learning trials; 2) Total recall, indexing the sum of the words recalled, either free or cued, in the three immediate recall trials; 3) Total Delayed Free Recall, indexing the delayed free recall; 4) Total Delayed Recall, indexing the total amount of words recalled, either free or cued, in the delayed recall trial.

***Repeatable Battery for the Assessment of Neuropsychological Status (RBANS) in the EPAD cohort***

The story memory and story recall subtests of the RBANS (Randolph et al., 1998) assess the individual's ability to encode, store, and retrieve verbal information, providing insights into aspects of episodic memory and overall cognitive functioning. The examiner reads a brief narrative or story to the individual being assessed. After the story is read, the individual is asked to immediately recall as much information as possible about the story. The examiner scores the individual's responses based on specific criteria, including the accuracy and completeness of the recall. Approximately 20 to 25 minutes after the Story Memory subtest, the examiner asks the individual to recall the same story again. Similar to the Story Memory subtest, the individual is prompted to provide as much detail as possible about the story. The examiner scores the individual's recall based on specific criteria, assessing the accuracy and completeness of the information provided.

***Supplementary statistical analyses***

In addition to the LME modelling, we determined the impact of CSF biomarkers on longitudinal hippocampal subfields volume (HSV) and cognitive performance using linear regression conducted on their difference score between consecutive visits (v2-v1). As performed for the voxel-wise analyses describe above, we first inspected the effect of AT status by conducting an analysis of variance (ANOVA) and adjusting for the effects of baseline age, sex *APOE*-ε4, years of education, follow-up time and TIV. In case of a significant main effect of AT status, Bonferroni-corrected post-hoc tests were inspected to retrieve differences in pairwise comparisons. Subsequently and in separate models, we entered the effects of continuous CSF biomarker concentrations along with the covariates defined above. In these set of analyses, results were considered significant if surviving a false discovery rate (FDR)-corrected p-value of p<0.05. In the ALFA study cohort, all statistical models were additionally adjusted by CAIDE risk scores and WMH.

**Supplementary results**

***Impact of CSF biomarkers on the hippocampal subfields***

We found significant main effects of AT status on the right dentate gyrus (DG) (F_2,282_=3.81, p=0.023), entorhinal cortex (EC) (F_2,282_=3.83, p=0.023) and subiculum (SUB) (F_2,282_=3.88, p=0.023). Bonferroni-adjusted post-hoc comparisons revealed significantly higher GMV atrophy rates in the A+T- compared to the A-T- group in the right DG (p=0.034; A-T-: 2.21% [95% CI 1.41 to 3.01]; A+T-: 4.24% [95% CI 2.77 to 5.71]) and right SUB (p=0.038; A-T-: 1.68% [95% CI 0.97 to 2.41]; A+T-: 3.34% [95% CI 2.05 to 4.63]), while the A+T+ showed reduced GMV in the right EC compared to the A-T- subgroup (p=0.02; A-T-: 2.01% [95% CI 0.89 to 3.12]; A+T+: 5.78% [95% CI 2.52 to 0.05]). When modeling CSF biomarkers continuously, CSF Aβ42/40 significantly predicted GMV atrophy in the bilateral CA1, as well as the right DG, SUB, and right BA35 (Supplementary Table 15). For CSF p-tau181 and NfL no significant effects were observed. When repeating the analyses in the A+T- individuals, we confirmed a significant main effect of CSF Aβ42/40 on the right DG (F_1,64_ = 4,91, p=0.03), right EC (F_1,64_ = 7,01, p=0.01), right BA35 (F_1,64_ = 8,03, p=0.006), as well as the left CA1 (F_1,64_ = 4,86, p=0.03) (Supplementary Table 16).

**Impact of CSF markers on longitudinal cognition using a regression approach**

In the ALFA sample, we found a significant main effect of AT stages on the longitudinal PACC trajectory (F_2,296_=5.99, p=0.003). Bonferroni-corrected post-hoc tests revealed that, compared with A-T-, both A+T- (p=0.029) and A+T+ (p=0.016) showed a steeper PACC decline over time. In addition, a significant main effect of AT was found on the total recall index of the FCSRT (F_1,316_ = 4.93; p=0.008). Bonferroni-corrected post-hoc tests revealed that the A+T+ showed significantly higher decline compared to both the A-T- (p=0.006) and the A+T- (0.028). When modelling CSF biomarkers continuously, baseline CSF Aβ42/40 predicted longitudinal decline in PACC (F_1,300_=14.21, p<0.001, pFDR=0.001), as well as total recall (F_1,316_ = 5.49; p=0.02, pFDR=0.04) and delayed free recall (F_1,316_ = 4.63; p=0.03, pFDR=0.04). No significant main effect on differential scores could be observed for CSF p-tau181 (Supplementary figure 5). In EPAD, a significant main effect of AT status was observed for both immediate (F_1,351_ =10.74, P<0.001, pFDR=0.002) and delayed recall (F_1,351_=7.88, P<0.001, pFDR=0.003). Bonferroni corrected post-hoc comparisons revealed that, compared with A-T-, the A+T- showed a significant decline both in immediate (p<0.001) as well as delayed recall (p=0.001). When modelling CSF biomarkers continuously, CSF Aβ42 significantly predicted longitudinal decline in immediate recall (F_1,351_=10.78, p<0.001, pFDR=0.003) and delayed recall (F_1,351_=4.39, P=0.023, pFDR=0.032). As observed in the ALFA sample, no significant main effect on differential scores could be observed for CSF p-tau181 (Supplementary figure 6). Results of the analyses conducted in the A+T- subgroup in each study sample yielded no significant results.

**Supplementary Table 1 – Sample’s characteristics in the ALFA cohort**

|  | **A-T- (n=215)** | **A+T- (n=88)** | **A+T+ (n=27)** | **p-value** |
| --- | --- | --- | --- | --- |
| Age, y | 60.20 (4.50) | 61.58 (5.07) | 63.66 (4.29) | 0.001 |
| Sex, f | 128 (59.5%) | 49 (55.7%) | 18 (66.7%) | 0.58 |
| Education, y | 13.71 (3.42) | 13.70 (3.56) | 11.70 (3.51) | 0.02 |
| *APOE*-ε4 carrier, n | 91 (42.3%) | 72 (81.8%) | 14 (51.9%) | 0.001 |
| CSF Aβ42/40* | 0.086 (0.008) | 0.053 (0.01) | 0.044 (0.01) | <0.001 |
| CSF p-tau181 (log10)* | 1.12 (0.13) | 1.17 (0.12) | 1.49 (0.09) | <0.001 |
| CSF NfL (log10)* | 1.861 (0.14) | 1.91 (0.13) | 2.04 (0.11) | <0.001 |
| TIV | 1426.02 (154.49) | 1457.66 (144.57) | 1387.16 (160.23) | 0.08 |
| WMH* | 3266.66 (450.73) | 3421.75 (299.48) | 3948.06 (450.34) | 0.86 |
| CAIDE score | 5.632 (1.65) | 5.71 (1.70) | 6.01 (1.33) | 0.739 |

Data are presented as mean (SD) or n (%); y = years; f = female; CSF = cerebrospinal fluid; WMH = white matter hyperintensities. *ANOVA adjusting by Age, Sex, *APOE*-e4 and years of education

**Supplementary Table 2 – Sample’s characteristics in the EPAD cohort**

|  | **A-T- (n=234)** | **A+T- (n=97)** | **A+T+ (n=28)** | **p-value** |
| --- | --- | --- | --- | --- |
| Age, y | 65.61 (6.21) | 66.05 (7.27) | 71.27 (6.04) | 0.001 |
| Sex, f | 125 (53.4%) | 55 (56.7%) | 12 (57.1%) | 0.43 |
| Education, y | 14.26 (3.61) | 14.30 (3.73) | 12.96 (4.39) | 0.19 |
| *APOE*-ε4 carrier, n | 66 (28.2%) | 38 (39.2%) | 19 (67.9%) | 0.001 |
| CSF Aβ42* | 1625.09 (392.04) | 710.83 (188.51) | 716.13 (187.61) | <0.001 |
| CSF p-tau181 (log10)* | 1.19 (0.11) | 1.15 (0.15) | 1.56 (0.09) | <0.001 |
| TIV | 1487.31 (152.76) | 1499.45 (141.87) | 1482.61 (138.06) | 0.73 |

Data are presented as mean (SD) or n (%); y = years; f = female; CSF = cerebrospinal fluid. *ANOVA adjusting by Age, Sex, *APOE*-e4 and years of education

**Supplementary Table 3 – Differences in longitudinal GMV changes between AT stages subgroups in the ALFA cohort**

|  |  | **MNI coordinates** | | |  |  |  |
| --- | --- | --- | --- | --- | --- | --- | --- |
| **Contrast** | **Region** | **x** | **y** | **z** | **t-value** | **Cluster size** | **p-value** |
| **A+T- < A-T-** | Right Inferior Temporal | 62 | -27 | -27 | 2.92 | 123 | 0.002 |
|  | Right Middle Temporal | 52 | -63 | 6 | 2.91 | 85 | 0.002 |
|  | Right Orbitofrontal | 4 | 21 | -27 | 3.72 | 327 | <0.001 |
|  | Right Parahippocampal | 22 | -22 | -21 | 3.12 | 79 | 0.001 |
|  | Left Posterior hippocampus | -20 | -38 | 2 | 2.85 | 51 | 0.002 |
| **A+T+ < A-T-** | Right Fusiform | 40 | -62 | -15 | 4.12 | 796 | <0.001 |
|  | Left Fusiform | -38 | -22 | -21 | 3.27 | 94 | 0.001 |
|  | Right inferior temporal | 48 | -16 | -28 | 3.69 | 591 | <0.001 |
|  | Right hippocampus | 34 | -27 | -12 | 3.38 | 490 | <0.001 |
|  | Right amygdala | 21 | -4 | -20 | 3.05 | 73 | 0.001 |
| **A+T+ < A+T-** | Right fusiform | 40 | -62 | -15 | 3.42 | 215 | <0.001 |
|  | Right hippocampus | 36 | -26 | -14 | 2.97 | 59 | 0.002 |

A=Aβ status; T=p-tau status; MNI=Montreal Neurological Institute

**Supplementary table 4 – Main effects of AT status in the ALFA cohort, in models adjusted by while matter hyperintensities and cardiovascular risk factors**

| **Contrast** | **t-value** | **Cluster size [k]** | **Brain region** |
| --- | --- | --- | --- |
| **A+T- < A-T-** |  |  |  |
|  | 3.668456 | 327 | Right orbitofrontal |
|  | 3.343784 | 127 | Left inferior frontal |
|  | 3.0643 | 267 | Right inferior frontal |
|  | 3.02782 | 212 | Right anterior insula |
|  | 2.959778 | 101 | Right middle temporal |
|  | 2.835087 | 135 | Right inferior temporal |
|  | 3.13 | 100 | Right hippocampus |
| **A+T+ < A-T-** |  |  |  |
|  | 4.066573 | 774 | Right fusiform |
|  | 3.621808 | 596 | Right inferior temporal |
| **A+T+ < A+T-** |  |  |  |
|  | 3.372229 | 199 | Right fusiform |
|  |  |  |  |

A=Aβ status; T=p-tau status;

**Supplementary Table 5 – Differences in longitudinal GMV changes between AT stages subgroups in the EPAD cohort**

|  |  | **MNI coordinates** | | |  |  |  |
| --- | --- | --- | --- | --- | --- | --- | --- |
| **Contrast** | **Region** | **x** | **y** | **z** | **t-value** | **Cluster size** | **p-value** |
| **A+T- < A-T-** | Left fusiform | -33 | -44 | -18 | 3.09 | 79 | 0.001 |
|  | Left middle temporal | -67 | -27 | -12 | 2.96 | 102 | 0.002 |
|  | Left angular | -40 | -60 | 54 | 2.91 | 68 | 0.002 |
| **A+T+ < A-T-** |  |  |  |  |  |  |  |
|  | Left parahipocampal | -22 | -22 | -27 | 3.71 | 206 | <0.001 |
|  | Left middle temporal | -62 | -32 | -8 | 3.56 | 1710 | <0.001 |
|  | Right fusiform | 32 | -74 | -16 | 3.13 | 154 | 0.001 |
| **A+T+ < A+T-** |  |  |  |  |  |  |  |
|  | Left inferior temporal | -63 | -40 | -26 | 3.63 | 168 | <0.001 |
|  | Right parahipocampal | 28 | -21 | -30 | 3.19 | 106 | 0.001 |

A=Aβ status; T=p-tau status; MNI=Montreal Neurological Institute

**Supplementary table 6 - Main effects of continuous CSF AD biomarkers in the ALFA cohort, in models adjusted by while matter hyperintensities and cardiovascular risk factors**

| **Predictor** | **t-value** | **Cluster size [k]** | **Brain region** |
| --- | --- | --- | --- |
| ***CSF Aβ42/40*** |  |  |  |
|  | 4.85 | 3215 | Right inferior temporal |
|  | 4.27 | 1165 | Left hippocampus |
|  | 4.21 | 518 | Right entorhinal |
|  | 3.97 | 215 | Left lingual |
|  | 3.60 | 386 | Right inferior temporal |
| ***CSF p-tau*** |  |  |  |
|  | 3.65 | 439 | Left anterior insula |
|  | 3.43 | 358 | Left hippocampus |
|  | 3.43 | 104 | Right hippocampus |
|  | 3.320 | 277 | Left superior occipital |
|  | 3.06 | 121 | Right amygdala |
|  | 3.03 | 156 | Left putamen |

**Supplementary table 7 - Differences in longitudinal cortical thickness (CTh) changes between AT stages subgroups in the ALFA cohort**

|  |  | **MNI coordinates** | | |  |  |  |
| --- | --- | --- | --- | --- | --- | --- | --- |
| **Contrast** | **Region** | **x** | **y** | **z** | **t-value** | **Cluster size** | **p-value** |
| **A+T- < A-T-** | ITG | 63 | -29 | 0 | 2.85 | **27** | 0.002 |
|  | MTG | 46 | -54 | 20 | 2.87 | **13** | 0.002 |
| **A+T+ < A-T-** | MTG | 48 | -47 | 18 | 2.91 | 21 | 0.002 |
| **A+T+ < A+T-** | FG | -34 | -35 | -26 | 2.74 | 17 | 0.003 |

A=Aβ status; T=p-tau status; **ITG:** Inferior Temporal Gyrus**; MTG:** Middle Temporal Gyrus**;** FG: Fusiform Gyrus

**Supplementary table 8 - Main effects of continuous CSF AD biomarkers on longitudinal cortical thickness (CTh) change, in the ALFA cohort (entire sample)**

|  |  | **MNI coordinates** | | |  |  |  |
| --- | --- | --- | --- | --- | --- | --- | --- |
| **Predictor** | **Region** | **X** | **y** | **z** | **t-value** | **Cluster size** | **p-value** |
| **CSF Aβ42/40** | PHG | 37 | -32 | -18 | 2.74 | 28 | 0.002 |
| **CSF p-tau** |  |  |  |  |  |  |  |
|  | MTG | 48 | -58 | 13 | 3.95 | 149 | <0.001 |
|  | FG | -31 | -35 | -25 | 2.98 | 25 | 0.002 |
|  | ITG | 57 | -54 | 11 | 2.93 | 37 | 0.005 |
| **CSF NfL** | STG | -57 | -21 | 2 | 3.91 | 69 | <0.001 |
|  | SOG | 26 | -76 | 38 | 3.12 | 89 | 0.001 |
|  | AG | 34 | -68 | 43 | 2.85 | 18 | 0.002 |

PHG=Parahippocampal gyrus; MTG=Middle temporal gyrus; FG=Fusifgorm gyrus; ITG=Inferior temporal gyrus; STG=Superior temporal gyrus; SOG=Superior occipital gyrus; AG=Angular gyrus

**Supplementary table 9 - Main effects of continuous CSF AD biomarkers on longitudinal cortical thickness (CTh) change, in the ALFA cohort (Subset of A+T- only)**

|  |  | **MNI coordinates** | | |  |  |  |
| --- | --- | --- | --- | --- | --- | --- | --- |
| **Predictor** | **Region** | **x** | **y** | **z** | **t-value** | **Cluster size** | **p-value** |
| **CSF Aβ42/40** | **MTG** | -61 | -43 | -3 | 3.22 | **78** | 0.001 |
|  | ITG | 59 | -49 | -16 | 3.17 | 35 | 0.001 |
|  | TP | 52 | 9 | -34 | 2.92 | 17 | 0.002 |
|  | PCN | -7 | -69 | 29 | 2.89 | 33 | 0.002 |
| **CSF NfL** |  |  |  |  |  |  |  |
|  | IOG | -16 | -99 | -9 | 3.31 | 70 | 0.001 |
|  | MCC | -3 | -20 | 38 | 3.13 | 59 | 0.001 |

PCN=Precuneus; ITG=Inferior temporal gyrus; ITG=Inferior temporal gyrus; IOG=Inferior occipital gyrus; MCC=Middle cingulate cortex

**Supplementary table 10 - Differences in longitudinal cortical thickness (CTh) changes between AT stages subgroups in the EPAD cohort**

|  |  | **MNI coordinates** | | |  |  |  |
| --- | --- | --- | --- | --- | --- | --- | --- |
| **Contrast** | **Region** | **x** | **y** | **z** | **t-value** | **Cluster size** | **p-value** |
| **A+T+ < A-T-** | SPG | 31 | -40 | 44 | 4.26 | 289 | <0.001 |
|  | PHG | -31 | -24 | -27 | 3.82 | 148 | <0.001 |
|  | ITG | -48 | -74 | 12 | 3.50 | 109 | <0.001 |
|  | IFG | -36 | 47 | -12 | 3.74 | 73 | <0.001 |
|  | PCN | -8 | -53 | 37 | 4.03 | 354 | <0.001 |
| **A+T+<A+T-** | SPG | 31 | -39 | 43 | 4.37 | 252 | <0.001 |
|  | ITG | -38 | 46 | -13 | 4.15 | 106 | <0.001 |
|  | FG | 27 | -69 | -6 | 3.96 | 89 | <0.001 |
|  | MTG | -50 | -53 | 20 | 3.74 | 424 | <0.001 |
|  | PHG | -30 | -17 | -32 | 3.73 | 123 | <0.001 |

SPG=Superior parietal gyrus; PHG=Parahippocampal gyrus; ITG=Inferior temporal gyrus; IFG=Inferior frontal gyrus; PCN=Precuneus; FG=Fusiform gyrus

**Supplementary table 11 - Main effects of continuous CSF AD biomarkers on longitudinal cortical thickness (CTh) change, in the EPAD cohort (entire sample)**

|  |  | **MNI coordinates** | | |  |  |  |
| --- | --- | --- | --- | --- | --- | --- | --- |
| **Predictor** | **Region** | **x** | **y** | **z** | **t-value** | **Cluster size** | **p-value** |
| **CSF Aβ42** | AG | -36 | -58 | 38 | 3.51 | 169 | <0.001 |
|  | ITG | 45 | 2 | -36 | 3.31 | 39 | 0.001 |
|  | PHG | -29 | -25 | -28 | 3.17 | 79 | 0.001 |
|  | AG | 35 | -49 | 38 | 3.09 | 70 | 0.001 |
|  | PCN | -8 | -56 | 42 | 3.05 | 50 | 0.001 |
| **CSF p-tau** | ITG | -46 | -18 | -36 | 3.53 | 66 | <0.001 |
|  | IFG | 41 | 39 | 4 | 3.39 | 75 | <0.001 |
|  | AG | 32 | -38 | 41 | 3.19 | 64 | 0.001 |
|  | FG | -24 | -67 | 2 | 3.11 | 57 | 0.001 |
|  | IFG | -41 | 43 | -14 | 3.04 | 52 | 0.001 |

AG=Angular gyrus; ITG=Inferior temporal gyrus; PHG=parahippocampal gyrus; PCN=Precuneus; IFG=Inferior temporal gyrus; FG=Fusiform gyrus

**Supplementary table 12 - Main effects of continuous CSF AD biomarkers on longitudinal cortical thickness (CTh) change, in the EPAD cohort (Subset of A+T- only)**

|  |  | **MNI coordinates** | | |  |  |  |
| --- | --- | --- | --- | --- | --- | --- | --- |
| **Predictor** | **Region** | **x** | **y** | **z** | **t-value** | **Cluster size** | **p-value** |
| **CSF Aβ42** | MFG | 38 | 40 | 8 | 3.71 | 167 | <0.001 |
|  | IFG | 38 | 17 | 31 | 3.52 | 94 | <0.001 |
|  | PHG | 31 | -42 | -10 | 3.17 | 66 | 0.001 |
|  | INS | -29 | 29 | 3 | 3.13 | 45 | 0.001 |
|  | AG | 55 | -49 | 27 | 3.12 | 48 | 0.001 |
|  | PCN | -12 | -56 | 61 | 2.98 | 21 | 0.002 |
| **CSF p-tau** |  |  |  |  |  |  |  |
|  | IFG | 39 | 38 | 7 | 3.24 | 34 | 0.001 |
|  | STG | 47 | -17 | 0 | 3.05 | 31 | 0.001 |
|  | ITG | 56 | -33 | -8 | 3.05 | 28 | 0.001 |

MFG=Middle frontal gyrus; IFG=Inferior frontal gyrus; PHG=Parahippocampal gyrus; INS=Insula; AG=Angular gyrus; PCN=Precuneus; STG=Superior temporal gyrus

**Supplementary table 13 – Main effects of continuous AD biomarkers on hippocampal subfields using a linear regression approach**

|  | **Right CA1** | **Left CA1** | **Right DG** | **Right BA35** | **Right SUB** |
| --- | --- | --- | --- | --- | --- |
| Age | 8.85 (<0.01) | 1.85 (0.17) | 3.81 (0.05) | 1.59 (0.21) | 0.75 (0.38) |
| Sex | 0.21 (0.65) | 0.61 (0.43) | 2.15 (0.14) | 0.18 (0.67) | 1.44 (0.23) |
| Education | 1.21 (0.27) | 1.44 (0.23) | 0.01 (0.93) | 0.09 (0.76) | 0.43 (0.51) |
| *APOE*-ε4 carrier | 0.41 (0.52) | 0.56 (0.45) | 0.15 (0.69) | 2.11 (0.14) | 1.47 (0.22) |
| CSF Aβ42/40 | **9.62(<0.01)*** | **7.47 (<0.01)*** | **7.12 (<0.01)*** | **8.08 (<0.01)*** | **6.52 (0.01)*** |
| CSF p-tau181 | 4.55 (0.03) | 0.16 (0.68) | 0.31 (0.58) | 0.08 (0.77) | 1.53 (0.21) |
| CSF NfL | 0.38 (0.53) | 0.56 (0.45) | 2.29 (0.13) | 0.35 (0.55) | 3.48 (0.06) |
| Follow-up time | 2.89 (0.09) | 4.67 (0.03) | 0.31 (0.57) | 0.21 (0.64) | 0.17 (0.68) |

Data are presented as F-statistics (p-value); *FDRp<.05; Aβ=beta-amyloid; *APOE* = Apolipoprotein E; BA35=Brodmann area 35; CA1 = Cornu Ammonis 1; CSF = cerebrospinal fluid; DG = Dentate gyrus; NfL=Neurofilament light chain; p-tau181=phosphorylated tau; SUB=subiculum

**Supplementary Table 14 -** **Main effects of CSF Aβ42/40 on hippocampal subfields in the A+T- ALFA subsample using a linear regression approach**

|  | **Right SUB** | **Left ERC** | **Right BA35** |
| --- | --- | --- | --- |
| Age | 0.11 (0.73) | 0.91 (0.34) | 3.35 (0.07) |
| Sex | 7.08 (0.01) | 0.89 (0.34) | 2.62 (011) |
| Education | 0.63 (0.42) | 0.09 (0.76) | 0.21 (0.64) |
| *APOE*-ε4 carrier | 0.38 (0.54) | 0.23 (0.62) | 0.11 (0.74) |
| CSF Aβ42/40 | **13.29 (<0.01)** | **4.32 (0.04)** | **8.32 (<0.01)** |
| CSF p-tau | 0.03 (0.87) | 0.41 (0.52) | 0.11 (0.74) |
| CSF NfL | 3.31 (0.07) | 3.09 (0.08) | 2.23 (0.14) |
| Follow-up time | 0.01 (0.91) | 2.81 (0.09) | 0.19 (0.65) |

Data are presented as F-statistics (p-value); *APOE* = Apolipoprotein-E; BA36=Brodmann area 36; CSF = cerebrospinal fluid; ERC = Entorhinal cortex; SUB = Subiculum

**Supplementary Table 15 – Baseline AT status significantly predicted cognitive decline over time, in the ALFA sample**

|  |  |  |  |  |  |
| --- | --- | --- | --- | --- | --- |
| **Interaction term** | **Cognitive test** | **Param. Estimate** | **SEM** | **t-value** | **p-value** |
| **AT status * Time**  **(A-T- vs. A+T-)** | PACC | -0.04 | 0.01 | -2.96 | 0.003 |
| **AT status * Time**  **(A-T- vs. A+T+)** | PACC | -0.07 | 0.01 | -3.01 | 0.002 |
| **AT status * Time**  **(A-T- vs. A+T+)** | FCSRT-TR | -0.18 | 0.05 | -3.51 | <0.001 |

A=β-Amyloid status; T=p-tau status; PACC= Preclinical Alzheimer Cognitive Composite; FCSRT-TR=Total recall index of the Free and Cued Selective Reminding Test

**Supplementary Table 16 – Baseline CSF Aβ42/40 predicts cognitive decline over time in the ALFA sample**

|  |  |  |  |  |  |
| --- | --- | --- | --- | --- | --- |
| **Interaction term** | **Cognitive test** | **Param. Estimate** | **SEM** | **t-value** | **p-value** |
| **CSF Aβ42/40 * Time** | PACC | 1.54 | 0.39 | 3.94 | <0.001 |
| **CSF Aβ42/40 * Time** | FCSRT-TR | 1.98 | 0.83 | 2.37 | 0.018 |
| **CSF Aβ42/40 * Time** | FCSRT-DFR | 2.45 | 1.01 | 2.44 | 0.015 |

**PACC=** Preclinical Alzheimer Cognitive Composite; FCSRT-TR=Total recall index of the Free and Cued Selective Reminding Test; FCSRT-DFR=Delayed free recall index of the Free and Cued Selective Reminding Test

**Supplementary Table 17 – Baseline AT status significantly predicted cognitive decline over time, in the EPAD sample**

|  |  |  |  |  |  |
| --- | --- | --- | --- | --- | --- |
| **Interaction term** | **Cognitive test** | **Param. Estimate** | **SEM** | **t-value** | **p-value** |
| **AT status * Time**  **(A-T- vs. A+T-)** | RBANS Story Memory | -1.96 | 0.41 | -4.79 | <0.001 |
| **AT status * Time**  **(A-T- vs. A+T-)** | RBANS Story Recall | -0.82 | 0.22 | -3.71 | 0.002 |

**Supplementary Table 18 – Baseline CSF Aβ42 predicted cognitive decline over time, in the EPAD sample**

|  |  |  |  |  |  |
| --- | --- | --- | --- | --- | --- |
| **Interaction term** | **Cognitive test** | **Param. Estimate** | **SEM** | **t-value** | **p-value** |
| **CSF Aβ42 * Time** | RBANS Story Memory | 1.080e-03 | <0.001 | 3.24 | 0.001 |
| **CSF Aβ42 * Time** | RBANS Story Recall | 3.516e-04 | <0.001 | 1.97 | 0.04 |

**
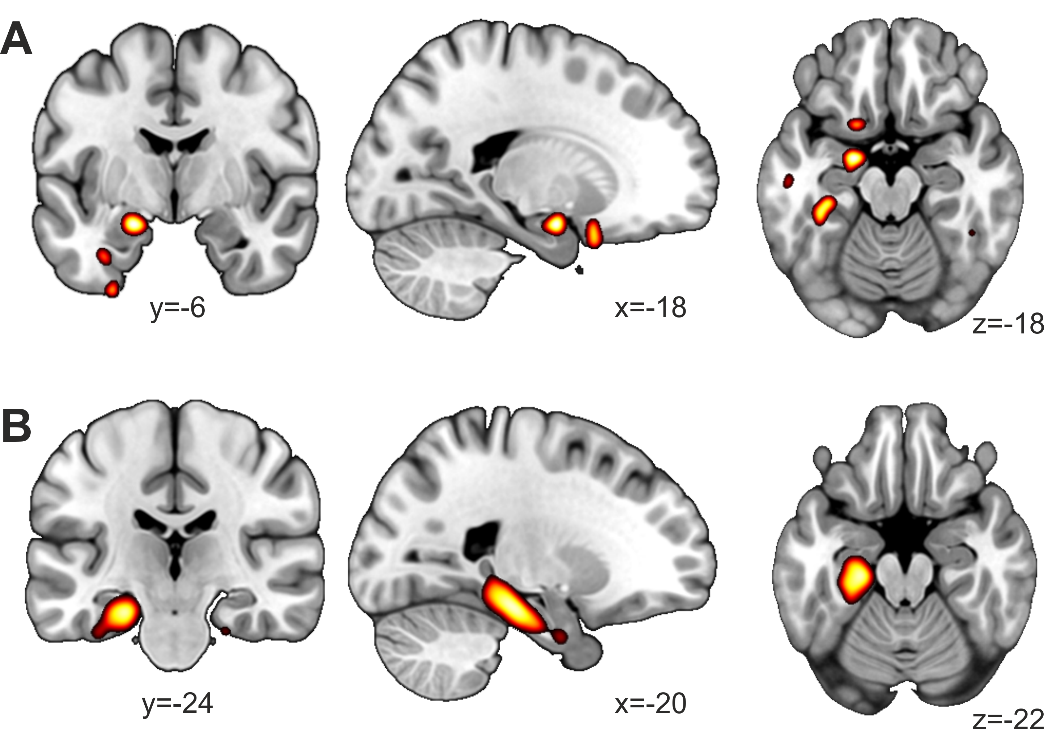
**

**
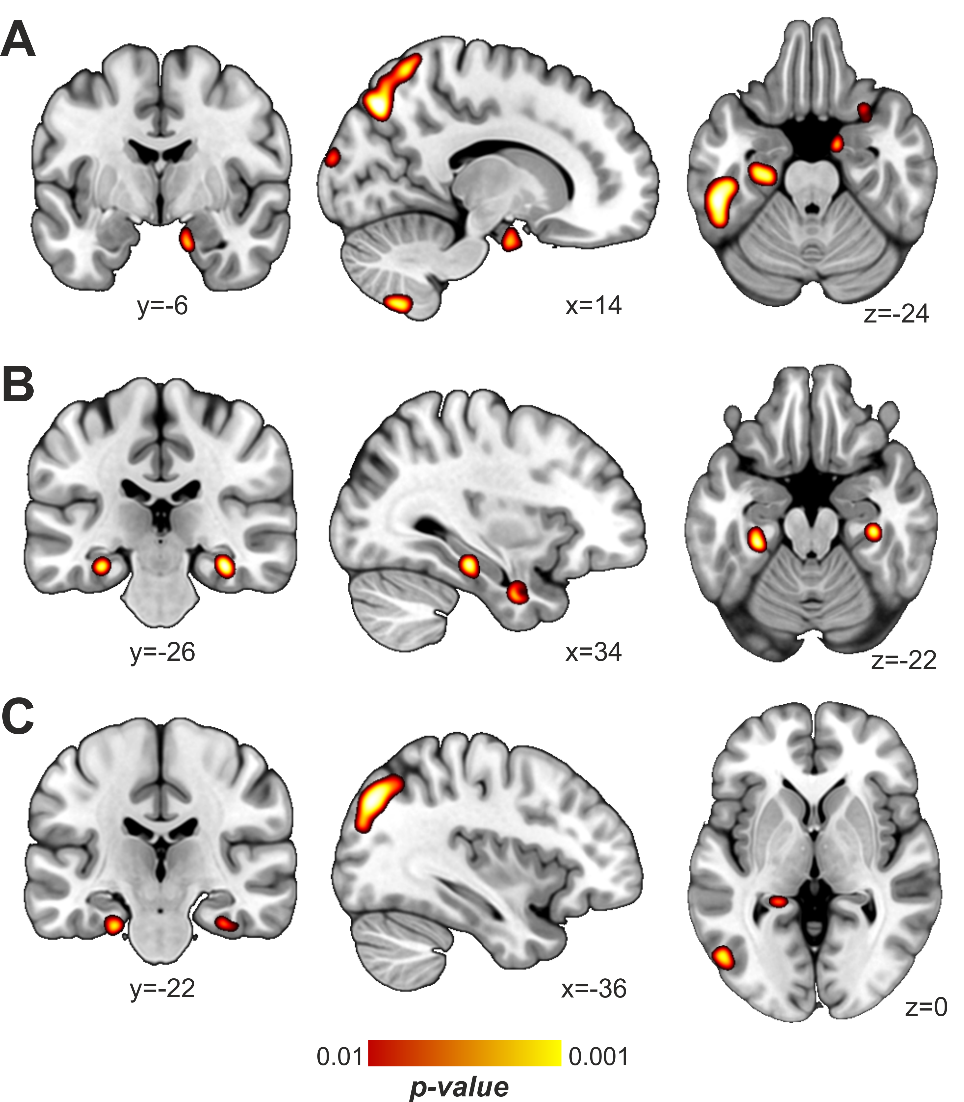
**

**Supplementary Fig. 1 – A)** Main effects of CSF Aβ42/40 in the ALFA cohort, restricted to the A+T- subsample. **B)** Main effects of CSF NfL in the ALFA cohort, restricted to the A+T- subsample. C) Main effects of CSF Aβ42 in the EPAD cohort, restricted to the A+T- subsample.

**
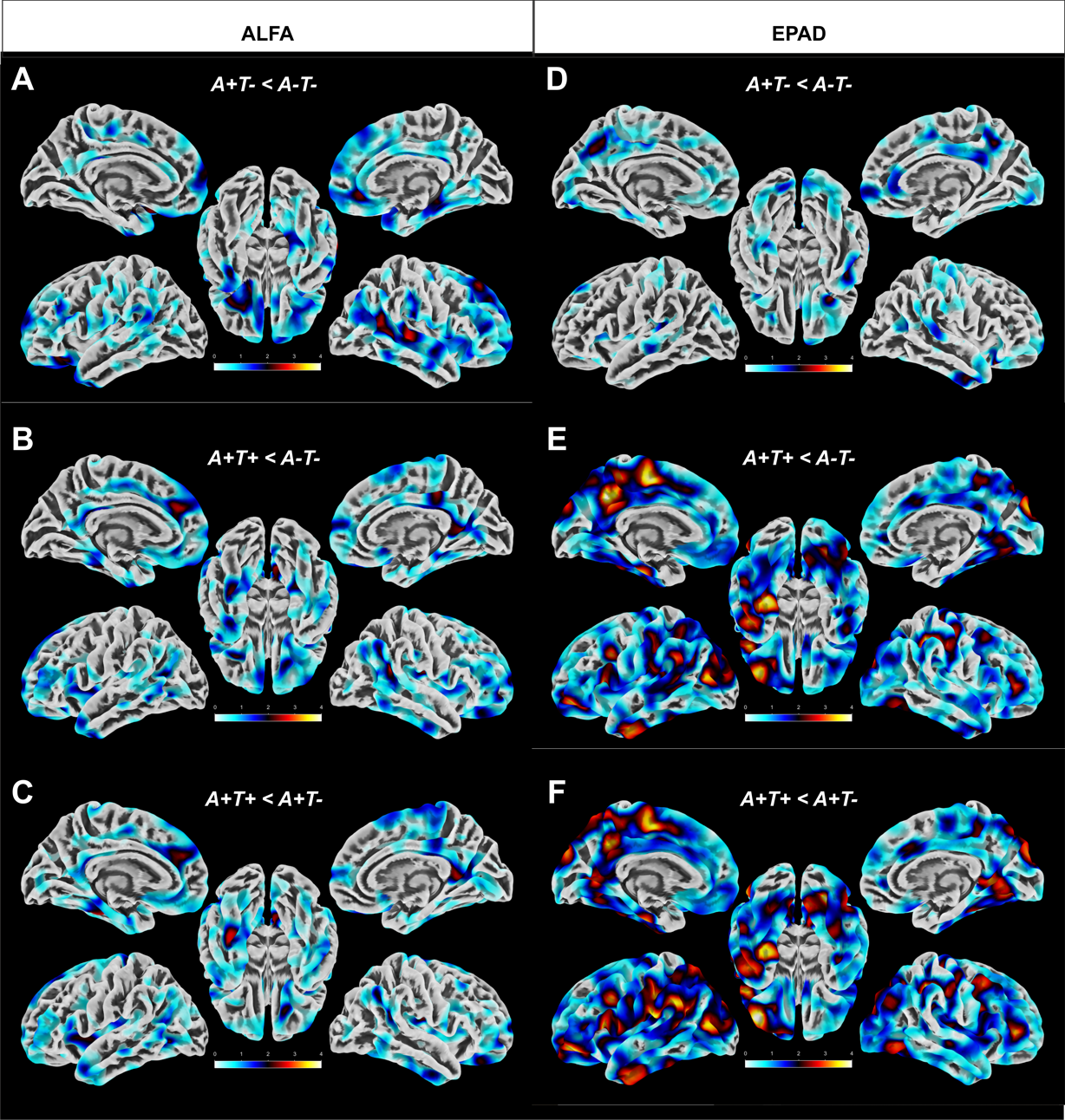
**

**Supplementary Fig.2 – Main Effect of AT status on longitudinal brain atrophy captured by cortical thickness maps**

A), B) and C) show surface rendering of longitudinal changed in cortical thickness for the three contrasts of interest in the ALFA sample. D), E) and F) illustrate the same contrasts calculated in the EPAD sample

**
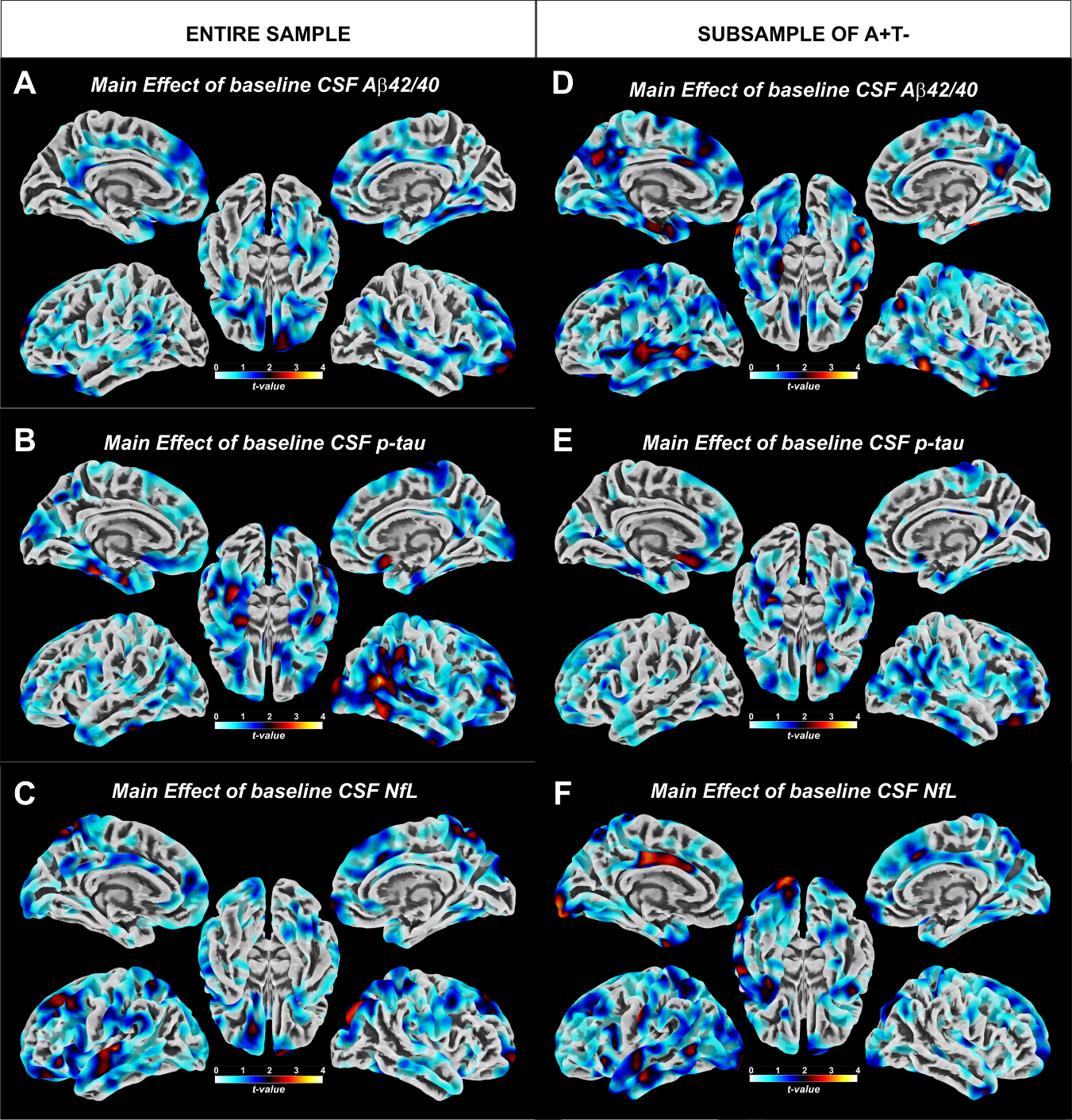
**

**Supplementary Fig.3 – Main Effect of continuous concentrations of baseline CSF AD biomarkers on longitudinal cortical atrophy as measured with cortical thickness in the ALFA sample.** Main effects of CSF Aβ42/40 (A), p-tau181 (B) and NfL (C) in the entire ALFA sample. D), E) and F) show main effects of the same biomarkers calculated within the subsample with positive Aβ42/40 biomarkers with no evidence of tau pathology (i.e., the A+T- subgroup)

**
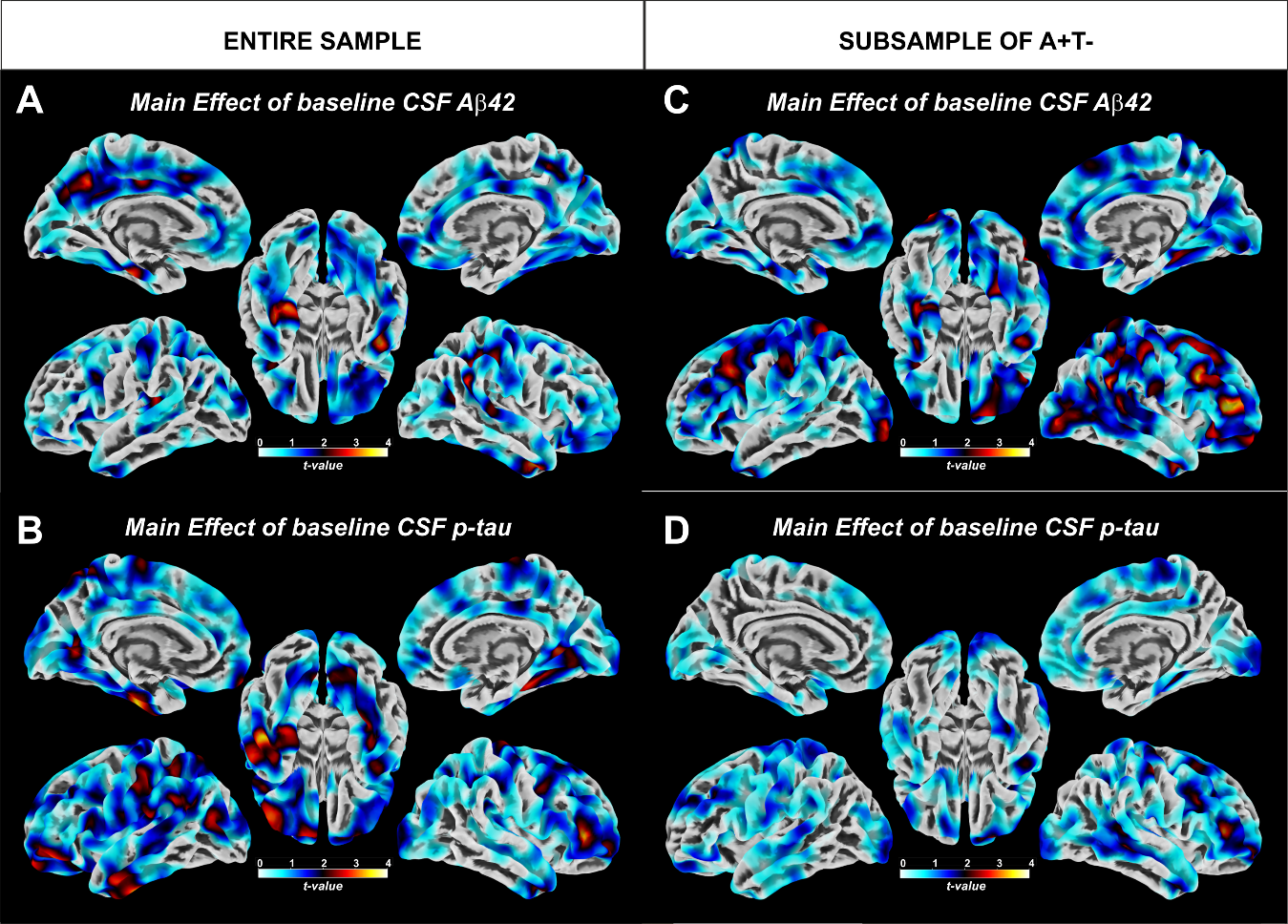
**

**Supplementary Fig.4 – Main Effect of continuous concentrations of baseline CSF AD biomarkers on longitudinal cortical atrophy as measured with cortical thickness in the EPAD sample.** Main effects of CSF Aβ42/ (A), and p-tau181 (B) in the entire EPAD sample. D) and E) show main effects of the same biomarkers calculated within the subsample with positive Aβ42 biomarkers with no evidence of tau pathology (i.e., the A+T- subgroup)

**
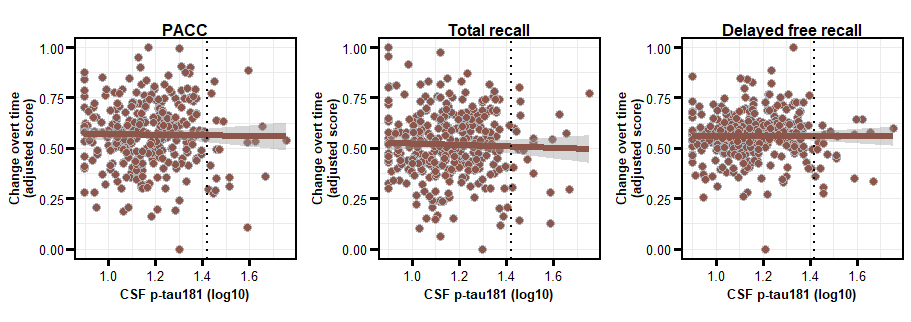
**

**Supplementary figure 5 –** Scatterplots showing the non-significant association between baseline CSF p-tau181 and changes in cognition, in the ALFA cohort. Dotted line indicates threshold for CSF p-tau181 positivity.

**
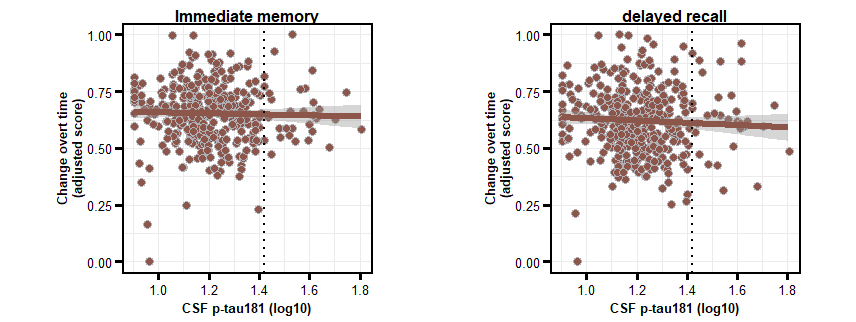
**

**Supplementary figure 6 –** Scatterplots showing the non-significant association between baseline CSF p-tau181 and changes in cognition, in the EPAD cohort. Dotted line indicates threshold for CSF p-tau181 positivity.

**Supplementary References**

Grau-Guinea L, Perez Enriquez C, Garcia-Escobar G, et al. Development, equivalence study, and normative data of version B of the Spanish-language Free and Cued Selective Reminding Test. *Neurologia (Engl Ed)* 2021; **36**(5): 353-60.

Kivipelto M, Ngandu T, Laatikainen T, Winblad B, Soininen H, Tuomilehto J. Risk score for the prediction of dementia risk in 20 years among middle aged people: a longitudinal, population-based study. *Lancet Neurol* 2006; **5**(9): 735-41.

Lorenzini L, Ingala S, Wink AM, et al. The Open-Access European Prevention of Alzheimer's Dementia (EPAD) MRI dataset and processing workflow. *Neuroimage Clin* 2022; **35**: 103106.

Randolph C, Tierney MC, Mohr E, Chase TN. The Repeatable Battery for the Assessment of Neuropsychological Status (RBANS): preliminary clinical validity. J Clin Exp Neuropsychol. 1998;20(3):310-9.

Sudre CH, Cardoso MJ, Bouvy WH, Biessels GJ, Barnes J, Ourselin S. Bayesian model selection for pathological neuroimaging data applied to white matter lesion segmentation. *IEEE Trans Med Imaging* 2015; **34**(10): 2079-102.
